# Advances in 4D-Printed Shape Memory Polymers from Materials, Mechanisms and Fabrication Techniques to Applications: A Comprehensive Review

**DOI:** 10.3390/ma19183869

**Published:** 2026-09-11

**Authors:** Gang Wang, Mengyao Dong, Junfang Shen, Xiangning Zhang, Meiling Du, Donglong Li, Kun Li, Xiaoli Zhang, Jingbo Chen

**Affiliations:** 1 Key Laboratory of Material Processing and Mold Technology, School of Mechanical Engineering, Chongqing Industry Polytechnic University, Chongqing 401120, China; wanggang@cqipu.edu.cn (G.W.); dongmy@cqipu.edu.cn (M.D.); xiangningzhang@cqipu.edu.cn (X.Z.); duml@cqipu.edu.cn (M.D.); dongldlme@163.com (D.L.); 2 School of Intelligent Manufacturing, Luoyang Institute of Science and Technology, Luoyang 471023, China; shen@lit.edu.cn; 3 School of Materials Science and Engineering, Zhengzhou University, Zhengzhou 450001, China; zhangxl@zzu.edu.cn (X.Z.); chenjb@zzu.edu.cn (J.C.)

**Keywords:** shape memory polymers, smart materials, actuation, 4D printing, excitation response

## Abstract

Shape memory polymers (SMPs) and their multifunctional composites have become important material systems for 4D printing because they can be processed into structures with programmed deformation and stimulus-responsive actuation. After being fixed in a temporary configuration, SMPs are able to recover their permanent shape when exposed to heat, electric or magnetic fields, and so on. This reversible shape change gives printed SMP structures functions beyond those of conventional static components, making them useful for biomedical devices, flexible electronics, and soft robotics. The performance of 4D-printed SMPs is determined not only by material chemistry but also by the design of the printed architecture and the manner in which external stimuli are applied. This review begins by covering the basic mechanisms that govern shape memory behavior in SMPs, including molecular switching, thermomechanical programming, and stimulus-controlled recovery. On this basis, representative printing methods for SMPs are compared, including stereolithography (SLA), fused deposition modeling (FDM), direct-write printing, and polymer inkjet printing. Recent applications are then considered in areas where programmed shape change has practical value, such as biomedical devices, soft robotics, and flexible electronics. The discussion also identifies unresolved problems in printing resolution, response speed, cyclic stability, structural design, and multifunctional coupling, which remain central barriers to wider use of 4D-printed SMP systems.

## 1. Introduction

The development of biology, medicine, and aerospace engineering fields has placed increasing demands on devices that are complex structures and capable of intelligent response. Conventional manufacturing methods, such as injection molding and extrusion, remain effective for large-scale production, but they are often constrained by mold design and processing routes and are generally more suitable for producing relatively simple geometries, including filaments, sheets, and tubes [1,2,3]. Additive manufacturing (referred to as 3D printing), as an emerging technology, allows the fabrication of complex three-dimensional structures without the need for molds [4,5,6,7,8,9]. However, the structures produced by 3D printing are typically static and lack adaptive behaviors when subjected to external stimuli, making it difficult to meet the need for intelligent and responsive devices.

Unlike 3D printing, 4D printing, first proposed by Tibbits, introduces time as an additional design variable [10]. This extra dimension (time) refers to the ability of 3D-printed structures to change their physical properties over time in response to external stimuli [11,12]. A crucial factor driving the success of 4D printing is the use of smart, shape-changing materials [13,14,15,16,17,18,19]. Research on these materials is inspired by the biomimetic behaviors observed in living organisms. A notable example is the Venus flytrap, whose trigger hairs can sense physical stimuli, prompting the plant to rapidly close its leaves in less than 100 milliseconds to capture prey [20]. SMPs are a class of smart materials capable of retaining a predefined shape. When deformed under specific conditions (such as the application of external force), they can spontaneously return to their original shape upon exposure to external stimuli, including heat, electricity, magnetism, or solvents [21,22,23,24]. The shape memory effect has been observed in polymers, such as amorphous polymers [25], semicrystalline polymers [26,27,28] and liquid crystal elastomers [29]. Compared with conventional polymers used in static structures, SMPs can undergo large recoverable deformation and their transition temperature and actuation mode can be tailored through molecular design or composite modification [30]. These characteristics make them attractive candidates for 4D-printed structures that require programmed shape change after fabrication [31,32,33]. Consequently, research on the 4D printing of SMPs is of paramount importance.

In this review, we begin by defining the shape memory effect and proceed to discuss recent developments in shape memory mechanisms. A deep understanding of the dynamics of polymer chains is crucial for comprehending phase transition processes and the overall regulation of shape memory behavior. We then explore different actuation methods for SMPs, including thermal, electrical, magnetic field, light and solvent stimuli. Subsequently, we discuss the methods of 4D printing, including stereolithography printing, fused deposition printing, direct-write printing and polymer jet printing and summarize the applications of 4D-printed SMPs. Finally, we address the future prospects and challenges associated with 4D printing of SMPs. Previous reviews have advanced the fields of 4D printing, soft-polymer additive manufacturing, shape memory polymers, and their composites from different perspectives (Table 1). However, these studies usually focus on a specific aspect of materials, processing, or applications, and rarely integrate shape memory mechanisms, thermomechanical foundations, external stimuli, additive manufacturing techniques, and application requirements within the specific framework of 4D-printed SMPs. In contrast, this review focuses on 4D-printed SMPs and is organized around the logic of shape memory mechanisms and thermomechanical foundations, stimulus-responsive actuation, additive manufacturing processes, representative applications, and current challenges and future directions. This review not only summarizes progress in the field, but also compares the applicable boundaries and trade-offs among different material systems, stimulus modes, and printing methods, with the aim of providing a clearer reference for the design of 4D-printed SMP devices in biomedical engineering, soft robotics, flexible electronics, and related fields.

## 2. Shape Memory Polymers and Composites

Shape memory polymers are a class of smart materials that retain an initial shape and, upon exposure to specific external stimuli (such as heat, light, electricity, magnetism, etc.), undergo deformation and become fixed in the altered shape [39]. When returned to the appropriate environmental conditions, these materials can autonomously revert to their original shape. This phenomenon is referred to as the shape memory effect. The process of shape memory programming represents the initial step in the shape memory cycle, during which the transformation pathway of the polymer is defined. The shape memory programming process of SMPs involves three key stages: shape deformation, shape fixation and the release of external stress. Before programming, the polymer must undergo deformation under the influence of external stress and heating. Elevated temperatures increase the polymer’s entropy, lower the energy barrier and facilitate the movement of polymer chains, making the polymer more manipulable. Once the polymer has been deformed into the desired temporary shape, it is fixed as the temperature decreases, with the deformation maintained by external stress. These conditions include physical and chemical changes that prevent the mobility of the polymer chain. After the external stress is released, the SMPs retain their temporary shapes until appropriate stimuli are applied, which trigger recovery to their original shapes.

SMPs are usually composed of a single polymer network, copolymer, or polymer blend, and their shape memory behavior mainly arises from the cooperation between fixed and reversible phases. This behavior is governed by glass transition temperature, melting temperature, crystallization behavior, crosslinking density, and segmental mobility. By contrast, SMPCs are composite systems formed by using an SMP as the matrix and introducing functional fillers, reinforcing fiber, nanoparticles, conductive networks, magnetic particles, or other responsive components. Therefore, while retaining the shape fixing and shape recovery capabilities of the matrix, SMPCs can further acquire mechanical reinforcement, electrical response, magnetic response, photothermal conversion, near-infrared response, or multi-stimulus responsiveness. The performance of SMPCs depends not only on the SMP matrix itself, but also strongly on filler type, content, particle size, dispersion state, interfacial bonding, orientation, and printing path.

### 2.1. Shape Memory Mechanism of SMPs

The shape memory effect observed in SMPs results from a programmed macroscopic shape change, facilitated by the unique polymer structure and intrinsic properties of the polymer. The polymer structure of SMPs consists primarily of two phases: a fixed phase and a reversible phase. The fixed phase consists of stable physical or chemical cross-links in the polymer network and maintains the permanent shape of the material. The reversible phase consisting of polymer segments within the polymer matrix enables shape transformation in response to external stimuli. These two phases synergistically drive the shape memory effect, enabling macroscopic shape changes.

At low temperatures, the polymer segments in the reversible phase of SMPs exist in a low-energy state, characterized by restricted mobility that effectively “freezes” the polymer chains. In this condition, the polymer macroscopically exhibits a glassy state, rendering it rigid and resistant to deformation. Upon heating above the polymer’s thermal transition temperature (*T*_trans_), these polymer segments gain energy, enhancing their mobility and enabling the polymer to transition macroscopically to a rubbery state. In this pliable state, the material becomes more deformable under external forces. When the temperature is subsequently lowered below the *T*_trans_ while maintaining the applied external force, the polymer segments in the reversible phase return to a “frozen” state and the deformation energy is stored within the polymer matrix. When reheated above its *T*_trans_, the stored energy is released, the polymer segments in the reversible phase revert spontaneously to their low-energy configuration [40].

### 2.2. Thermomechanical Theory of SMPs

The constitutive models for SMPs are generally classified into three main types: rheological models, phase transition models and hybrid models that combine both approaches [41,42]. The rheological model introduces viscosity into the description of shape memory behavior and can qualitatively reflect the viscoelastic and rate-dependent responses of SMPs. However, the model is based on one-dimensional, small-deformation, or linear viscoelastic assumptions, which limits the predictive capability for large deformation, multiaxial loading, and complex three-dimensional printed structures. In contrast, the phase transition model focuses on the glass transition and relates the thermal transition of the polymer to shape fixation and recovery. However, the model often simplifies broad glass transition regions, crystallization/melting processes, multiple transition temperatures, and heterogeneity in polymer networks. The hybrid model combines phase transition theory with viscoelastic theory, allowing both thermal transitions and time-dependent mechanical responses to be considered in the analysis of SMP behavior. However, hybrid models usually require many material parameters and experimental calibrations, and their predictive capability remains affected by material composition, loading path, stimulus conditions, and specimen geometry.

Tobushi et al. proposed a four-element model that incorporates a slippage mechanism [43]. In this model, internal friction is represented by a slippage element introduced into the linear viscoelastic equations. This formulation allows the thermo-viscoelastic response of SMPs to be described under one-dimensional small-deformation conditions. The stress–strain relationship is expressed as

(1) $$\overset{•}{\varepsilon }=\frac{\overset{•}{\sigma }}{E}+\frac{\sigma }{\mu }-\frac{\varepsilon -\varepsilon_{s}}{\lambda }+\alpha \overset{•}{T},\;\varepsilon_{s}=S\varepsilon_{c}$$

Here, *σ* represents stress, *ε* denotes strain, and *T* stands for temperature. Additionally, *E* refers to the elastic modulus, *α* represents the coefficient of thermal expansion and *λ* represents the relaxation time. The variables *ε*_c_ and *ε*_s_ correspond to the creep strain and residual strain, respectively. *S* represents the proportionality constant. Lin et al. adapted the four-element model to polyurethane SMPs by introducing a linear framework for describing shape memory behavior [44]. The model contains two parallel Maxwell branches: one represents the reversible phase, and the other represents the fixed phase. Because stress recovery was not included, however, its ability to predict the full shape memory response was limited. Tobushi further modified the original model by adding power-function nonlinear terms to both the elastic and viscous components, making the formulation more suitable for SMPs undergoing large deformation [45]. For particle-reinforced SMPCs, Yin developed a refined viscoelastic finite-deformation constitutive model based on the assumption that the composite system is homogeneous and isotropic. The model was used to describe the thermomechanical response and shape recovery behavior of SMPCs. In this formulation, a modified Adam–Gibbs model was coupled with a revised Eyring model to account for structural relaxation and yielding behavior [46]. Yang developed a physics-based constitutive model for amorphous SMPs that accounts for temperature and strain-rate effects [47]. The model treats SMPs as particle-reinforced composites, with the frozen phase serving as the matrix and the active phase as the dispersed particles. Using Mori-Tanaka theory, Yang derived the effective mechanical properties of the SMP and introduced an evolution equation for the frozen-phase volume fraction. Li et al. proposed a phase-transition-based thermodynamic constitutive model for amorphous SMPs, in which the evolution of frozen strain was treated as a time-dependent process [48].

### 2.3. Actuation Method for SMPs

Currently, SMPs are categorized according to the types of stimuli that trigger their response, including thermally activated SMPs, electrically activated SMPs, magnetically activated SMPs, photo-responsive SMPs and solution-driven SMPs.

#### 2.3.1. Heat-Driven SMPs

**Figure 1:**
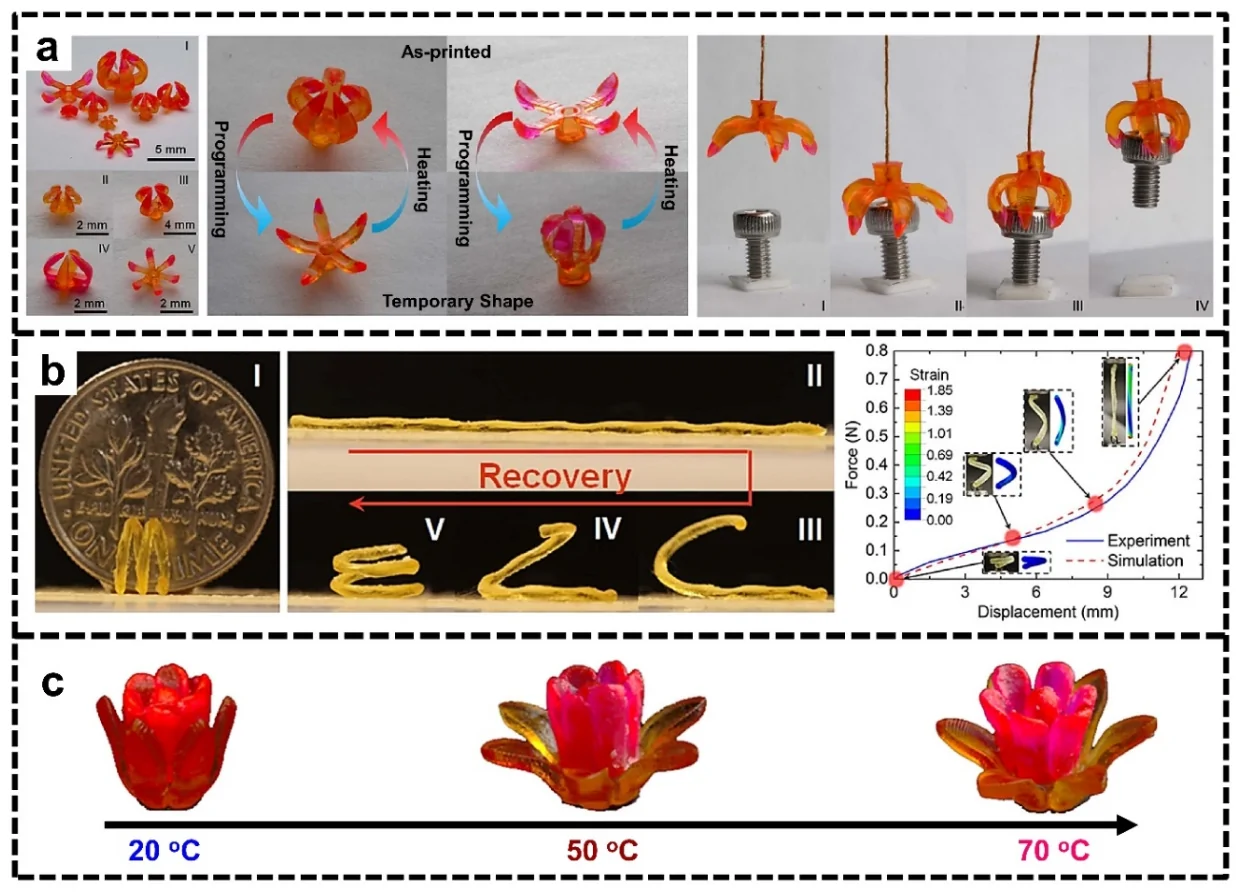
(**a**) The shape change between as-printed shape and temporary shape of multi-material grippers driven by heating and the snapshots of the process of grabbing an object. (**b**) The shape switching behavior of the spring from a straight strand temporary configuration to its original shape upon heating and the nonlinear deformation of the spring. (**c**) The sequential recovery of a multi-material flower driven by heating [54].

Thermally activated SMPs are used with temperature serving as the external stimulus, requiring a specific medium (gas, liquid, or solid) to transfer heat and enable rapid activation [49,50,51,52]. Wang et al. fabricated polycaprolactone/thermoplastic polyurethane (PCL/TPU) thermally activated shape memory composite filaments and investigated the influence of various compositions and printing parameters on the performance of these composites [53]. Ge et al. developed a novel SMP based on photopolymerized methyl methacrylate copolymer networks, demonstrating shape memory behavior in a multi-material system that transitioned from a fixed temporary shape to a recovered shape upon heating [54] (Figure 1a). Furthermore, they used an SMP with a *T*_g_ of 43 °C to create springs, examining their complex nonlinear large deformation shape memory behavior. It was observed that the springs became significantly stiffer as they approached their fully expanded state (Figure 1b). A multi-material flower with the inner and outer petals having different *T*_g_ values (inner petals with *T*_g_ = 56 °C, outer petals with *T*_g_ = 43 °C) was printed to demonstrate sequential shape recovery. Initially, all the petals were closed at 70 °C and subsequently cooled to 20 °C. After removing the external constraints, the flower was fixed in a temporary bud state, with both the inner and outer petals remaining closed. Sequential recovery was triggered by raising the temperature to 50 °C, causing only the outer petals to open. The inner petals opened later when the temperature reached 70 °C, resulting in the flower fully returning to its original open state (Figure 1c). Yang et al. developed a series of thermoplastic shape memory poly(ether-ether-ketone) (PEEK) materials that exhibited outstanding thermally activated shape memory effects, including high recovery rates (94–98.9%) and fixation rates (>99.5%) [55].

#### 2.3.2. Electrically Driven SMPs

Electrically driven SMPs are developed by incorporating conductive particles, such as carbon nanotubes, graphene, metals and carbon nanofibers, into the polymer matrix [56,57,58]. These SMPs exhibit electrical conductivity, enabling them to generate Joule heating when a voltage is applied. As the temperature rises and exceeds the material’s thermal transition temperature, the polymer undergoes spontaneous shape recovery. Although electrically driven SMPs are fundamentally thermally activated, the key distinction lies in their reliance on indirect heating through electrical energy, rather than direct thermal heating.

**Figure 2:**
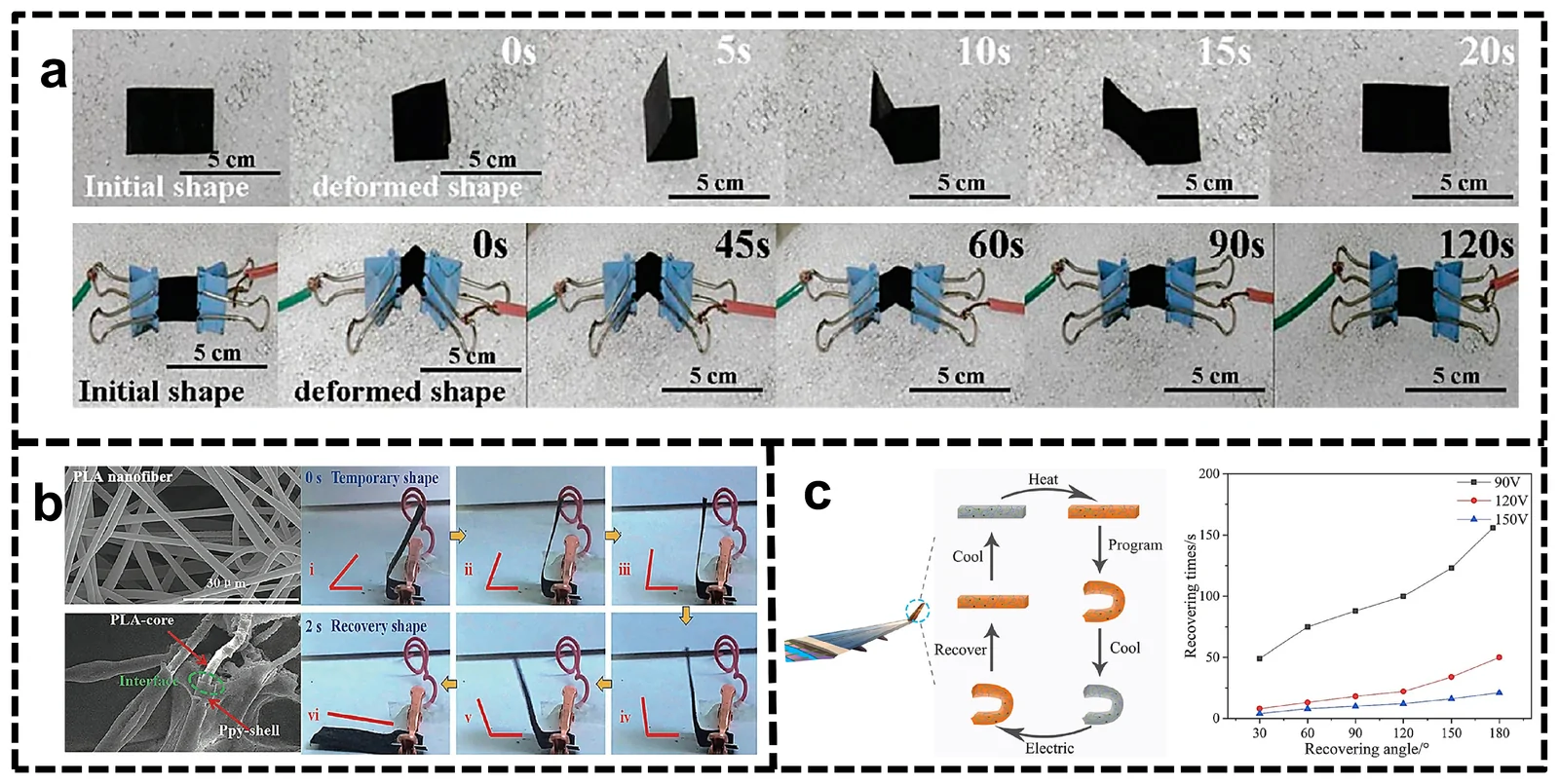
(**a**) Shape memory recovery process of the cPCL/PEG-M samples. Reproduced with permission from [59]. Copyright 2010, American Chemical Society. (**b**) Core–shell structure and electro-stimuli shape recovery behavior of PLA-polypyrrole fibers. Reproduced with permission from [60] Copyright 2018, American Chemical Society. (**c**) The process of the electroactive shape memory test (**left**) and the relationship between the shape recovery angle of HEP/CTBN/MA/CB composite skin (3 wt% CB) and the power-on time at different voltages (**right**). Reproduced with permission from [61]. Copyright 2022, John Wiley and Sons.

Xiao et al. fabricated one type of electroactive shape memory nanocomposite consisting of crosslinked poly(ε-caprolactone) (cPCL) and conductive multiwalled carbon nanotubes (MWCNTs). These nanocomposites exhibited excellent shape recovery both in 55 °C hot water and under a 50 V electric field [59] (Figure 2a). Zhang et al. further electrospun polylactic acid (PLA) into nanofiber membranes and subsequently synthesized PPy on the surface of these fibers via chemical vapor deposition at low temperatures. This process produced core–shell fibers, which provided enhanced conductive pathways [60] (Figure 2b). Ren et al. created composite deformable skins using a hydrogenated epoxy resin/terminal carboxylated butadiene acrylonitrile/maleic anhydride matrix, incorporating conductive carbon black [61]. With 2.6 wt% or 3 wt% carbon black, the samples could recover to 100% of their out-of-plane deformation shape under a 150 V electric field (Figure 2c). Zeng et al. developed 4D-printed samples using carbon fiber-reinforced polylactic acid (PLA) [62]. By applying an electric current, the samples demonstrated rapid recovery to their original shape within 75 s after programming. Liu et al. fabricated CNT/PLA filaments through physical blending and investigated the impact of printing direction on the material’s electrical conductivity, temperature distribution uniformity and shape recovery speed [63]. The samples printed in a combination of 0° and 90° directions exhibited more uniform temperature distribution and faster shape recovery. Additionally, a “trifurcated” device was printed, which allowed for selective and localized shape recovery through controlled current application.

#### 2.3.3. Magnetically Driven SMPs

Magnetically driven SMPs are fabricated by introducing magnetic particles, such as magnetite (Fe_3_O_4_), into the polymer matrix [64,65,66]. These SMPs represent a subclass of thermally activated shape memory materials. When subjected to an alternating magnetic field, the magnetic particles oscillate and generate heat through collisions or friction with the polymer matrix. Each magnetic particle acts as a micro-heat source and collectively, they generate sufficient thermal energy to elevate the material’s temperature [67,68,69,70].

**Figure 3:**
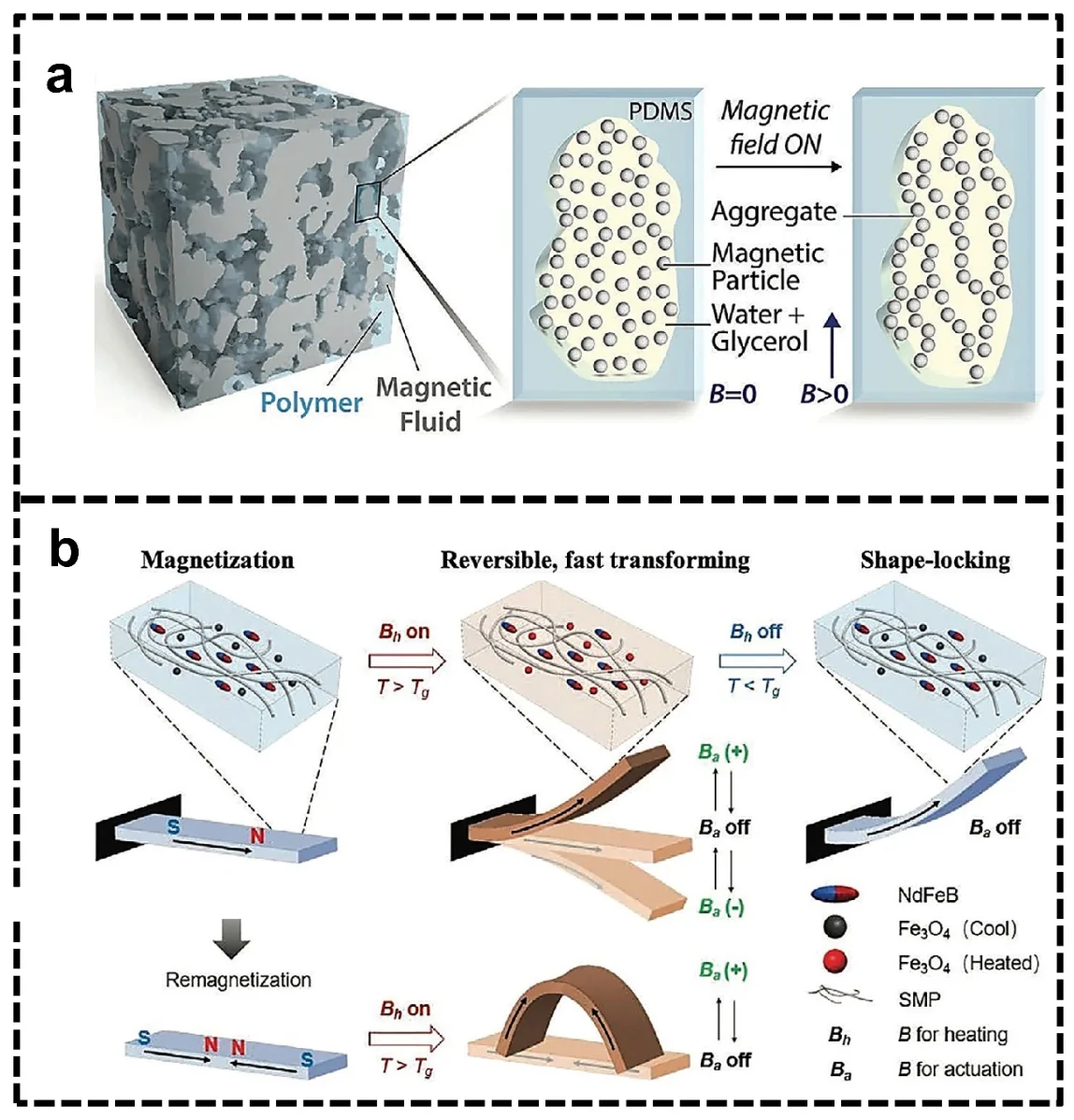
(**a**) 3D reconstruction of the internal two different phases inside the material obtained from tomography data. Reproduced with permission from [71]. Copyright 2019, John Wiley and Sons. (**b**) Working mechanism of magnetic shape memory polymers. The polymer is stiff at room temperature and it can be softened with a tunable shape under the magnetic field. Reproduced with permission from [72]. Copyright 2019, John Wiley and Sons.

Testa et al. prepared a soft magnetic shape memory composite that exhibited a nearly 30-fold increase in shear modulus and achieved reversible and rapid shape memory behavior [71] (Figure 3a). Ze et al. prepared an acrylate-based shape memory polymer (SMP) by embedding Fe_3_O_4_ and NdFeB microparticles, enabling sequential actuation through the adjustment of the respective particle loadings [72] (Figure 3b). Zhao et al. integrated magnetic Fe_3_O_4_ nanoparticles into PLA to fabricate porous, biomimetic tracheal scaffolds using fused deposition modeling [73]. These scaffolds exhibited excellent magnetic responsiveness, demonstrating reliable deformation capabilities and structural stability under magnetic fields. Wan et al. developed a bio-compatible quadruple shape memory composite by combining various material printing techniques with direct ink writing [74]. The composite, which incorporated Fe_3_O_4_ nanoparticles into a Poly(D,L-lactide-co-trimethylenecarbonate)/Poly(trimethylene carbonate) (PLMC/PTMC) matrix, exhibited a triple shape memory effect triggered by thermal stimuli. Furthermore, the Fe_3_O_4_ nanoparticles served as a magnetic-responsive component, enabling a fourth shape transformation in the matrix under an alternating magnetic field. Ye et al. used Fe_3_O_4_ nanoparticles as magnetic-responsive fillers in a PHB/PCL blend [75]. They found that the nanoparticles enhanced the fracture stress of the SMP and further investigated the effect of the cellulose nanofibers (CNFs) on the material’s magnetically driven shape recovery and overall mechanical performance. The printed snowflake model demonstrated rapid shape recovery from a temporary form to the original shape within 35 s under a magnetic field.

#### 2.3.4. Light-Driven SMPs

The construction of light-driven SMPs mainly follows two approaches. One approach involves the chemical modification of polymer chains by introducing photosensitive groups, resulting in intrinsic light-driven SMPs. These photosensitive groups can undergo reversible photochemical crosslinking reactions under ultraviolet (UV) light exposure, leading to changes in the conformations of the polymer chains, thereby triggering shape changes and exhibiting shape memory characteristics [76,77,78]. The second approach involves doping heat-driven SMPs with functional particles that have photothermal conversion effects, such as gold nanoparticles and carbon black. These particles absorb light energy and convert it into heat, thereby raising the temperature of the SMPs and activating their shape memory behavior. Light-driven SMPs offer the advantage of non-contact actuation and are easy to operate [79,80,81,82]. Compared to other types of driving mechanisms, light-driven SMPs can achieve precise point- or region-specific actuation and due to the nature of light, these materials are safer for human tissues [83]. Therefore, light-responsive SMPs hold broad application prospects in fields such as medicine, self-healing and microengineering.

**Figure 4:**
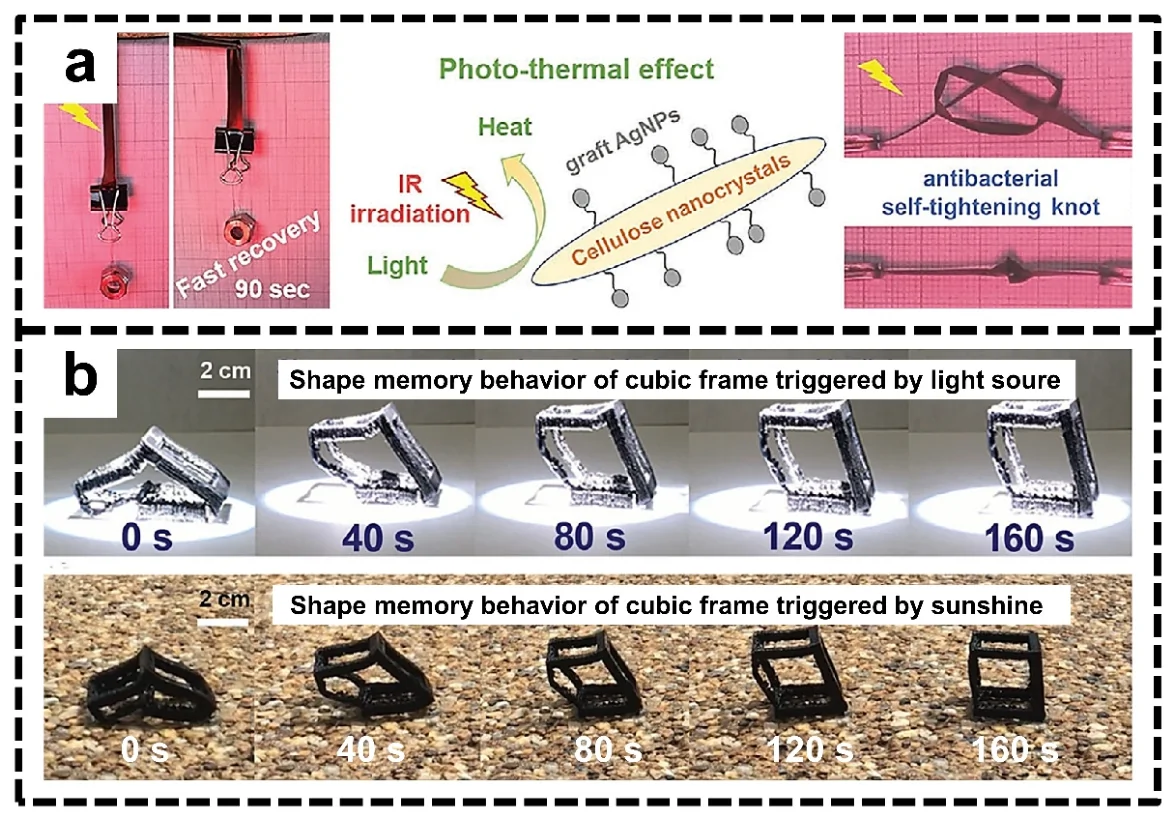
(**a**) Poly(ε-caprolactone) (PCL)-based shape memory polymer actuated upon infrared (IR) illumination. Reproduced with permission from [85]. Copyright 2018, American Chemical Society. (**b**) The shape recovering process of the cubic frame under 87 mW/cm^2^ of light source (**up**) and 76 mW/cm^2^ of sunshine (**down**). Reproduced with permission from [86]. Copyright 2017, John Wiley and Sons.

Fang incorporated organic rare-earth complexes into poly[ethylene-vinyl acetate] (PEVA) to produce selective near-infrared (NIR) light-responsive SMPCs [84]. Toncheva et al. grafted silver nanoparticles onto cellulose nanocrystals, creating an SMPC with rapid infrared response [85] (Figure 4a). Yang et al. fabricated PU/CB light-driven shape memory 3D devices, where the excellent photothermal conversion efficiency of carbon black (CB) allowed the printed cubic structure to almost completely recover from a flattened temporary shape to its original 3D form within 160 s under an 87 mW/cm^2^ light source and 76 mW/cm^2^ sunlight exposure [86] (Figure 4b). Kong introduced different light-responsive groups (azobenzene and cinnamic acid) to achieve multi-stage deformation under different wavelengths (254 nm and 365 nm) of light [87]. By adjusting shape fixation and programming with light exposure, they successfully realized diversified information transmission goals and demonstrated programmed deformation behaviors, such as “human” and “flower” transformations.

#### 2.3.5. Solution-Driven SMPs

**Figure 5:**
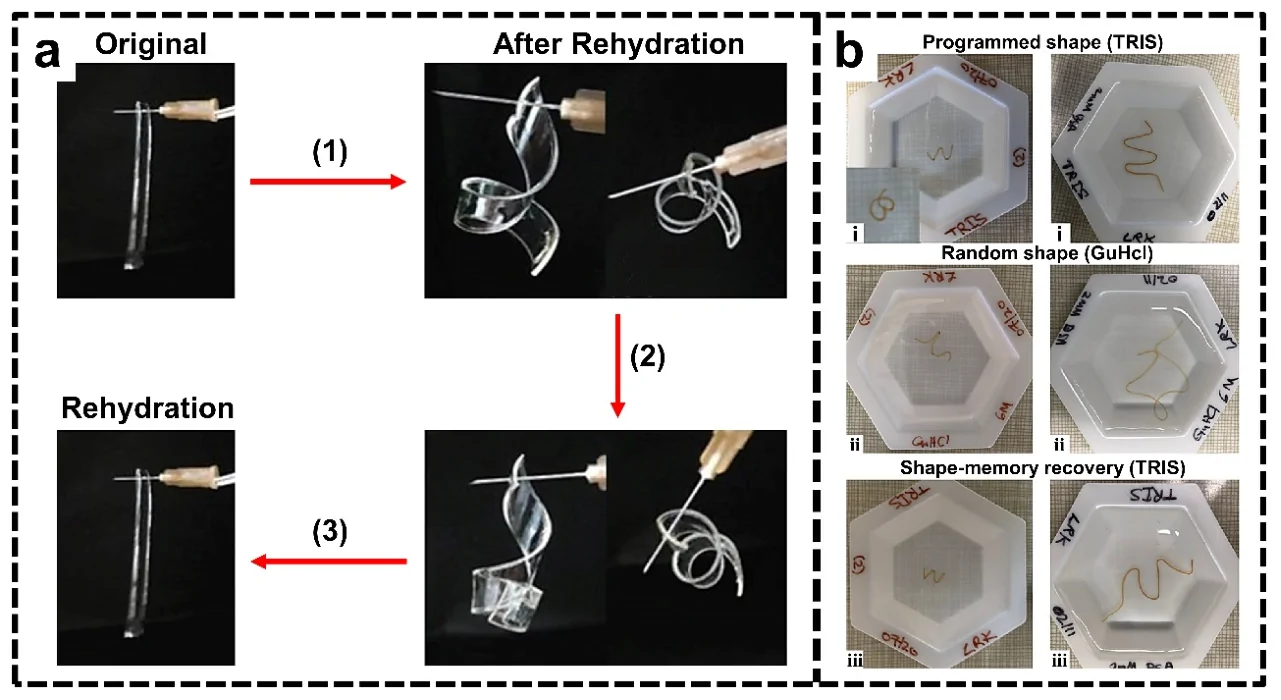
(**a**) Deformation process of Janus assembly containing hydrogel and hydrophilic PDMS during dehydration and rehydration [88]. (**b**) Shape memory cycle of protein hydrogel programmed as a spring (**left** **i–iii**) or W-shape (**right i–iii**) [89].

Solution-driven SMPs can undergo swelling when exposed to water, organic solvents and other substances. Small molecules such as water and organic solvents infiltrate the polymer chain network, acting as plasticizers and lowering the polymer’s thermal transition temperature. This reduction in transition temperature allows the shape memory effect of the polymer to be activated at lower temperatures. Liang et al. developed an intelligent hydrogel with high stretchability, self-healing and shape memory behavior [88]. The hydrogel is composed of a network structure of polyacrylamide and phenylboronic-acid-grafted alginate, with its shape memory behavior driven by dehydration and rehydration (Figure 5a). Khoury reported the shape memory effect of a protein hydrogel derived from bovine serum albumin [89]. The protein hydrogel can maintain a fixed programmed shape in a solution of tris(2,2′-bipyridyl) ruthenium(II) chloride. However, when transferred to a guanidine hydrochloride solution, the hydrogel exhibited a significant loss in hardness and shape recovery (Figure 5b). Melocchi prepared glycerol (GLY)-plasticized polyvinyl alcohol (PVA) filaments through physical blending using an extruder and subsequently employed fused deposition printing to create rectangular strips and U-shaped models based on the PVA filaments [90]. After programming the 3D-printed models into temporary shapes, they were immersed in water at room temperature and demonstrated near-complete recovery to their original shape within 120 min. Ren employed a dual-layer printing strategy to achieve a structure that responded to both thermal and water stimuli [91]. They printed a soft gripper with a double-layer structure, where the PU layer and shape memory polymer polyvinyl chloride (SMPVC) layer were printed at a 45° angle radially. The soft gripper could bend uniformly to grasp heavy objects under the dual stimuli of water, heat, or both.

We systematically compared thermal, electrical, magnetic, optical, and water/solution stimuli (Table 2). Overall, thermal stimulation offers broad material applicability and high shape fixity and recovery efficiency, but its response rate is affected by heat conduction and structural size, its spatial selectivity is relatively limited, and biomedical applications require strict control of the transition temperature to avoid thermal tissue damage. Electrical stimulation enables fast responses and locally programmable actuation through conductive networks, but high voltage, electrode contact, Joule heating, and the long-term safety of conductive fillers limit in vivo applications. Magnetic stimulation enables non-contact remote actuation, has favorable tissue penetration and potential biomedical advantages, and some magnetic composite SMPs can recover within tens of seconds. However, the biosafety of magnetic particles, safe ranges of field strength/frequency, and local overheating still need to be considered. Optical stimulation, especially near-infrared photothermal actuation, provides high spatial controllability and non-contact activation, but its efficiency depends on light penetration depth, light intensity, and filler distribution, whereas ultraviolet light has limited applicability in biomedical settings. Solution stimulation is generally milder and is suitable for body-fluid-related systems such as hydrogels, PVA, and biodegradable polymers, but its response is governed by water diffusion and swelling kinetics, with response times typically ranging from minutes to hours and with relatively limited output force and rapid actuation capability. Because response time and shape recovery efficiency vary greatly with material composition, specimen size, printed architecture, and testing conditions, these comparisons are intended as representative ranges and trends rather than direct rankings under identical experimental conditions.

## 3. Method for 4D Printing Shape Memory Polymers

SMP materials, as a typical class of stimulus-responsive materials, play a crucial role in the development of 4D printing. Currently, the primary techniques employed for 4D printing with SMPs include stereolithography (SLA), fused deposition modeling (FDM), direct ink writing (DIW) and inkjet printing.

### 3.1. Stereolithography Printing

Stereolithography (SLA) printing technology integrates photopolymerization with rapid prototyping techniques. It utilizes a liquid photosensitive polymer, which undergoes rapid photoinitiated polymerization upon exposure to UV light, causing a significant increase in molecular weight and transitioning the material from a liquid to a solid state. The process begins by creating a 3D model of the desired object using computer-aided design software, which is then divided into layers. Once the system is initialized, the worktable is positioned and a computer-controlled laser-curing source scans the resin in the liquid pool according to pre-generated code, following a “point-to-line, line-to-surface” approach. This causes the resin to cure and adhere to the worktable, forming the first layer of the object. After each layer is scanned, the areas not exposed to the laser remain in liquid form. The worktable then rises by the height of one layer and a fresh layer of liquid resin is applied. The next layer is scanned and adheres to the previous layer, continuing layer by layer until the final layer is scanned, completing the 3D object as per the design specifications.

**Figure 6:**
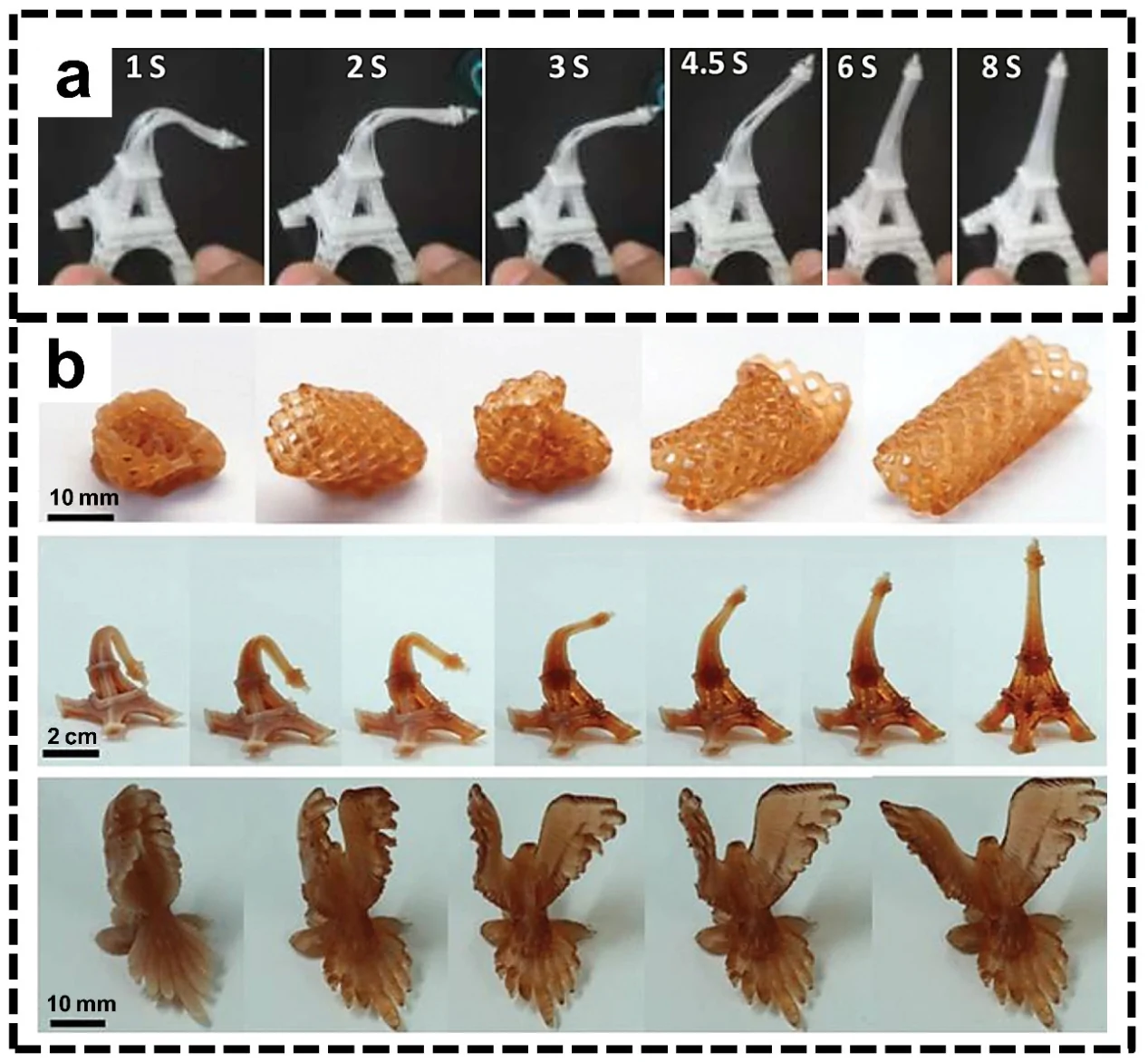
(**a**) The shape recovery process of the printed Eiffel Tower with a dryer. Reproduced with permission from [92]. Copyright 2017, American Chemical Society. (**b**) The shape memory of a model cardiovascular, an Eiffel Tower model and a bird model printed by SLA with the molten macromethacrylate. Reproduced with permission from [93]. Copyright 2015, John Wiley and Sons.

Yu et al. developed a photosensitive shape memory epoxy-ene-acrylate polymer by combining epoxy monomers with acrylate monomers [92]. Using SLA technology, they 4D-printed a shape memory epoxy acrylate “Eiffel Tower” structure, which could be further deformed into a temporary curved shape at 85 °C. When reheated to 85 °C using a heat gun, the material could actively return to its original printed form based on its shape memory effect (Figure 6a). Zarek successfully printed photosensitive polycaprolactone acrylate using a SLA printer. Under UV light exposure, the melt undergoes crosslinking, forming a network structure that enabled it to exhibit deformation behavior. The team printed several three-dimensional structures, such as scaffolds, eagles, and the Eiffel Tower and induced deformation under thermal stimulation [93] (Figure 6b). Zhao synthesized a polyurethane acrylate and used SLA molding to form SMP [94]. Through folding–unfolding tests and shape memory cycle measurements, the material demonstrated excellent shape memory properties, including high shape recovery rate, shape fixity, recoverability and outstanding durability. Zhang et al. incorporated polycaprolactone into methyl methacrylate-based SMPs and successfully fabricated high-resolution SMPs with dual-network self-healing characteristics using SLA. In the dual-network self-healing SMP system, the addition of 20% or more polycaprolactone restored the mechanical properties of damaged structures to over 90% of their original strength [95].

### 3.2. Fused Deposition Printing

Fused deposition modeling (FDM) is an extrusion-based shaping technique commonly employed with thermoplastic polymer materials [96]. These polymers, upon reaching their melting temperature, transition into a liquid state and subsequently solidify as they cool, facilitating a thermal-solid phase change. Before printing, the 3D model undergoes dimensional reduction, wherein the complex design is divided into multiple 2D layers using computer-aided design software. The thermoplastic filament is then melted in an extrusion head with a built-in heater and extruded through a nozzle. The movement of the extrusion head, precisely controlled by the software, allows the melted filament to be deposited layer by layer, each representing a cross-sectional slice of the final object. As the material cools, each deposited layer rapidly solidifies, thereby completing the formation of one cross-sectional slice. After one layer is finished, the worktable descends by one layer height, and the nozzle proceeds with scanning and extruding the second layer in the same manner, forming a new cross-sectional layer. Owing to the adhesive properties of the thermoplastic material, the newly extruded layer adheres securely to the previously formed layer. This layer-by-layer deposition process continues from the bottom to the top, with each successive layer bonding to the one beneath it, until the final layer is deposited. The result is a fully formed solid model or part [97,98,99,100]. Currently, FDM has been employed in 4D printing of thermoplastic shape memory polymers, post-crosslinked shape memory polymers and thermoplastic shape memory polymer composites [101]. Moreover, FDM can also be indirectly used for the 4D printing of some thermosetting shape memory polymers.

**Figure 7:**
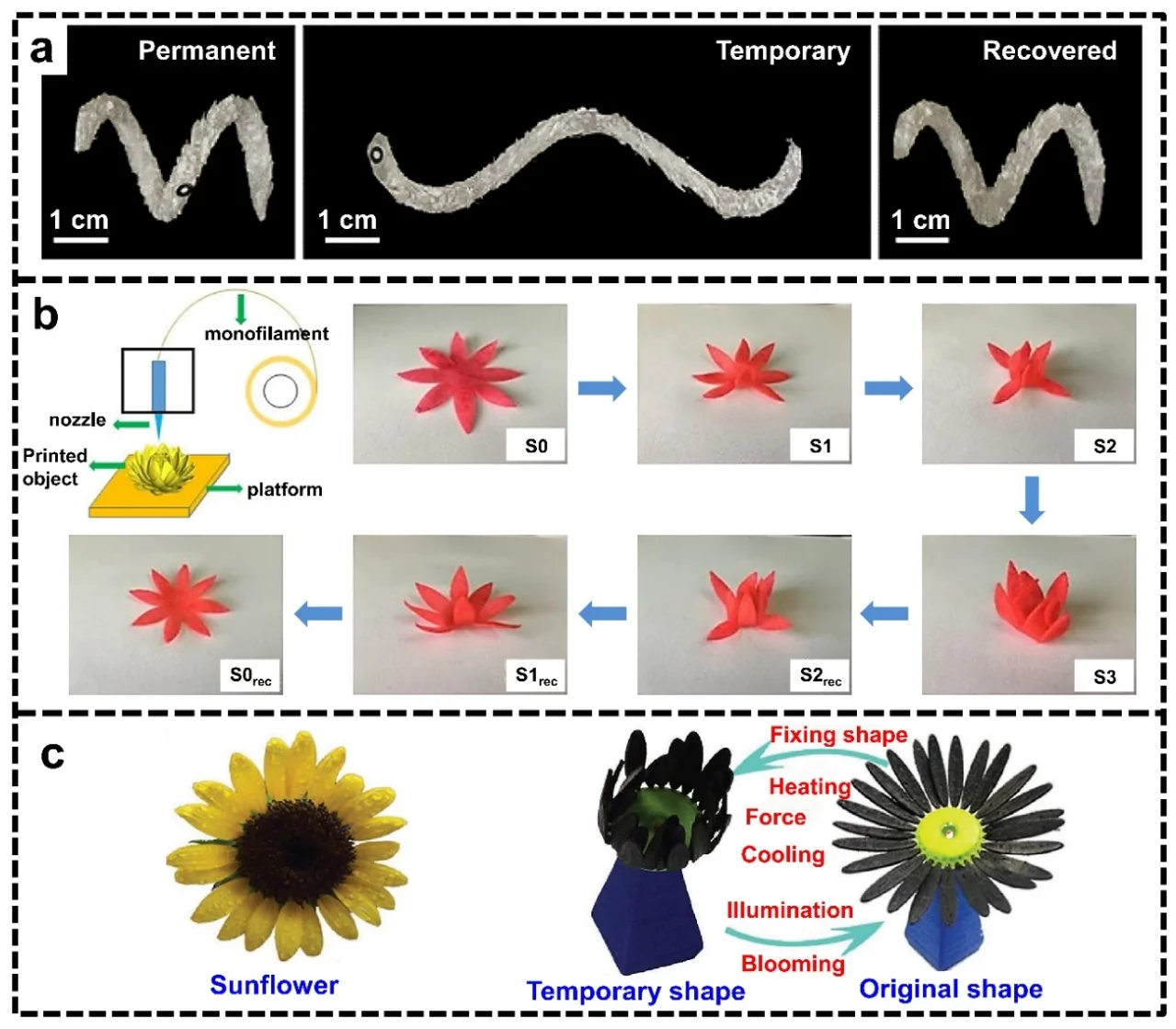
(**a**) Shape memory cycles for the printed spring from permanent to the temporary and to the recovered shape. Used with the permission of the American Chemical Society, from [Three-Dimensional Printed Shape Memory Objects Based on an Olefin Ionomer of Zinc-Neutralized Poly(ethylene-co-methacrylic acid), ACS Appl. Mater. Interfaces, 9 (32) (2017)] [102]. (**b**) Shape memory process of a 3D-printed flower with eight petals. Used with the permission of the Royal Society of Chemistry, from [3D printing of tunable shape memory polymer blends, Royal Society of Chemistry, 5(33) (2017)] [103]. (**c**) The real image of the sunflower (**left**) and photo-triggered shape memory behavior of the 3D printed sunflower (**right**). Reproduced with permission from [86]. Copyright 2017, John Wiley and Sons.

Zhao employed ionomeric polystyrene resin with significant shape memory behavior as the raw material to study the influence of FDM on the shape memory performance of printed products [102]. The results revealed that spring structures fabricated by FDM could undergo deformation and recovery over multiple cycles, exhibiting excellent shape memory properties (Figure 7a). Chen et al. prepared a thermoplastic polyethylene–styrene/ethylene/butene copolymer–polyethylene wax mixture with multiple shape memory effects through melt blending [103]. The multi-shape memory polymer material, printed into a star-shaped structure, was capable of memorizing three distinct temporary shapes at different temperatures. When exposed to a thermal field, the folded star structure could gradually unfold into its original shape (Figure 7b). Hua et al. used a PLA/MWCNT composite to print on paper substrates via FDM [104]. The PLA-based MWCNT composite printed by FDM exhibited a more refined structural advantage and superior photothermal response characteristics, making it a promising candidate for flexible photothermal-responsive actuators. Yang et al. combined FDM technology with photothermal SMPs to achieve 3D printing of photothermal-responsive shape memory components [86]. The composite material, consisting of thermoplastic PU as the matrix and CB as the photothermal conversion particles, was extruded into filaments using an extrusion machine. These filaments were then printed into structures such as scaffolds and flowers using a 3D printer. The structure, when in a temporary shape, actively returns to its initial form under temperature or sunlight stimuli (Figure 7c).

### 3.3. Direct-Write Printing

Direct ink writing (DIW) is a liquid-based printing technology that uses inks with a certain viscosity, commonly referred to as “printing inks”. Prior to printing, the ink is loaded into a high-pressure syringe, which is then mounted on a 3D movable platform. The syringe is controlled via computer-assisted software and it moves in accordance with the pre-converted 3D model code. Simultaneously, a pressure pump extrudes the ink onto the substrate. During the printing process, the ink may be solidified through various methods such as UV light irradiation, natural evaporation, or other curing techniques. Once a layer has solidified, the 3D movable platform is translated upward by one layer height to begin the formation and curing of the next layer. This process continues in a repetitive sequence until the final structure is completed [105,106]. The viscoelasticity properties of the ink are critical for the success of DIW printing. Nanoparticles can be introduced into the ink to achieve or improve its shear-thinning and thixotropic properties [107]. Additionally, in the DIW printing of polymer, the ink typically consists of a viscoelastic fluid. Even though the ink can maintain its printed shape on the substrate, post-processing methods are generally necessary to solidify the extruded ink and ensure the formation of a stable polymer structure. These post-processing techniques often include thermal curing [108] or UV polymerization [109].

**Figure 8:**
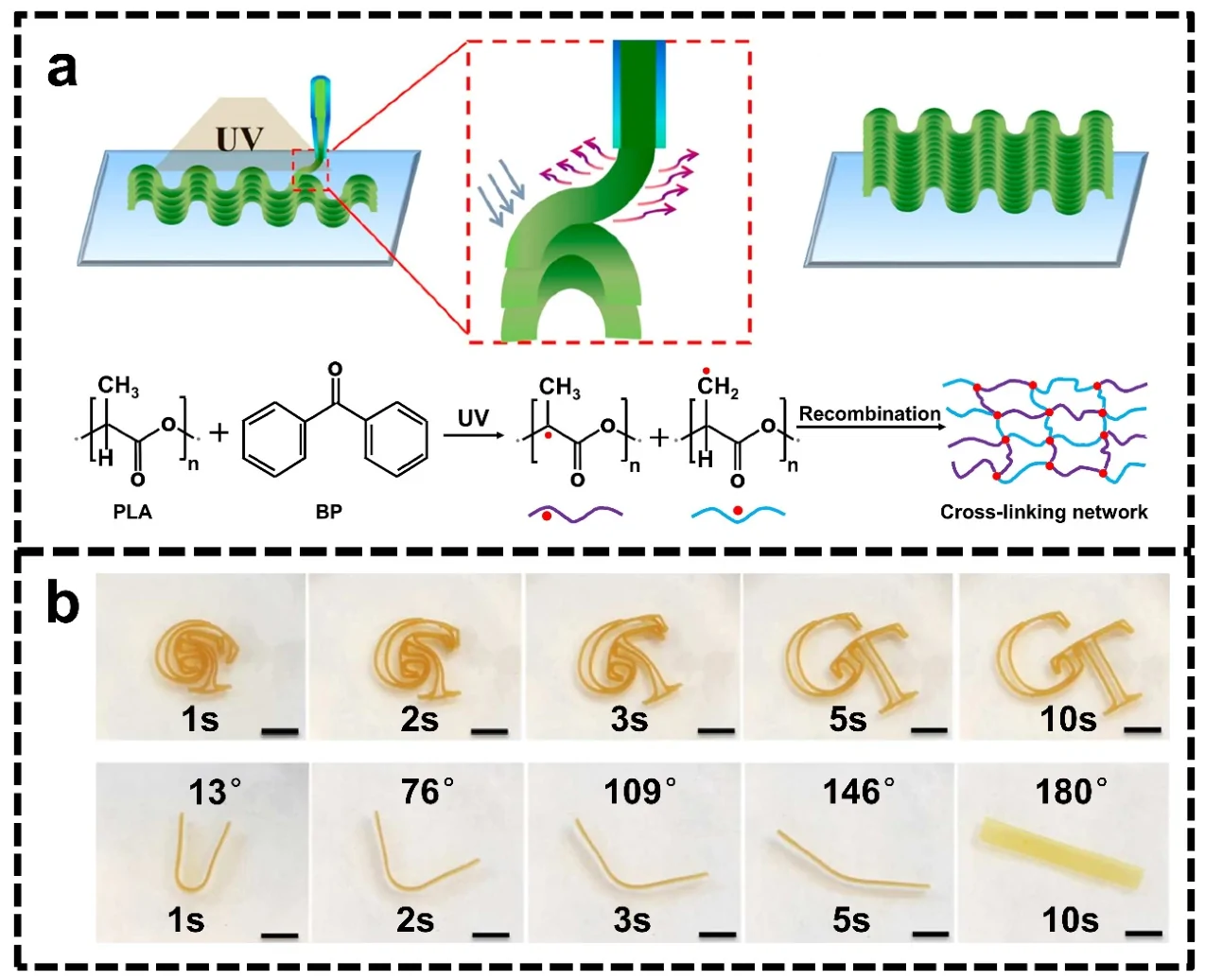
(**a**) Schematic illustration of the DIW printing of the 4D active shape-changing architecture. Reproduced with permission from [110]. Copyright 2017, American Chemical Society. (**b**) The shape memory for printed samples; the logo “GT” (**up**) and strip sample (**down**) in a 104 °C pump oil bath (scale bars in are 8 mm). Used with the permission of the Royal Society of Chemistry, from [Fabrication of tough epoxy with shape memory effects by UV-assisted direct ink write printing, Soft Matter, 14(10) (2018)] [108].

Wei et al. employed dichloromethane as a solvent to dissolve PLA, benzoin ketone and iron oxide nanoparticles, thereby creating a printable “ink” [110]. During the printing process of DIW, dichloromethane gradually evaporated and UV light was used to rapidly solidify the ink, forming a crosslinked network structure. This crosslinked network endowed the material with shape memory properties, enabling the successful fabrication of a magnetically responsive 4D structure (Figure 8a). Through adjustments in material composition, Gladman developed an ink with shear-thinning properties [111]. By altering the 3D printing parameters and incorporating nanofiber bundles into the material, they replicated intricate biomimetic complex structures. Chen adjusted the material composition to obtain a shear-thinning viscoelastic fluid “ink” [108]. Using DIW technology, they achieved 4D printing of epoxy-based SMPs composites. After the 4D-printed structure was formed, it could still undergo significant deformation under external force in an oil bath at 104 °C. Furthermore, the deformed shape could quickly and actively recover to its original shape when re-exposed to 104 °C after cooling and fixation (Figure 8b).

### 3.4. Polymer Jet Printing

The printing principle of polymer jetting technology closely resembles that of traditional inkjet printers, with the primary distinction being that polymer jetting employs photosensitive resins for the creation of 3D models, whereas inkjet printers typically use liquid inks for 2D pattern formation. The process of polymer jetting involves the ejection of photosensitive polymers onto a work platform via a printhead, followed by curing with UV light. During this process, the printhead and the light source move synchronously. Once one layer of the model is completed, the process is repeated for subsequent layers until the final 3D structure is formed [112].

**Figure 9:**
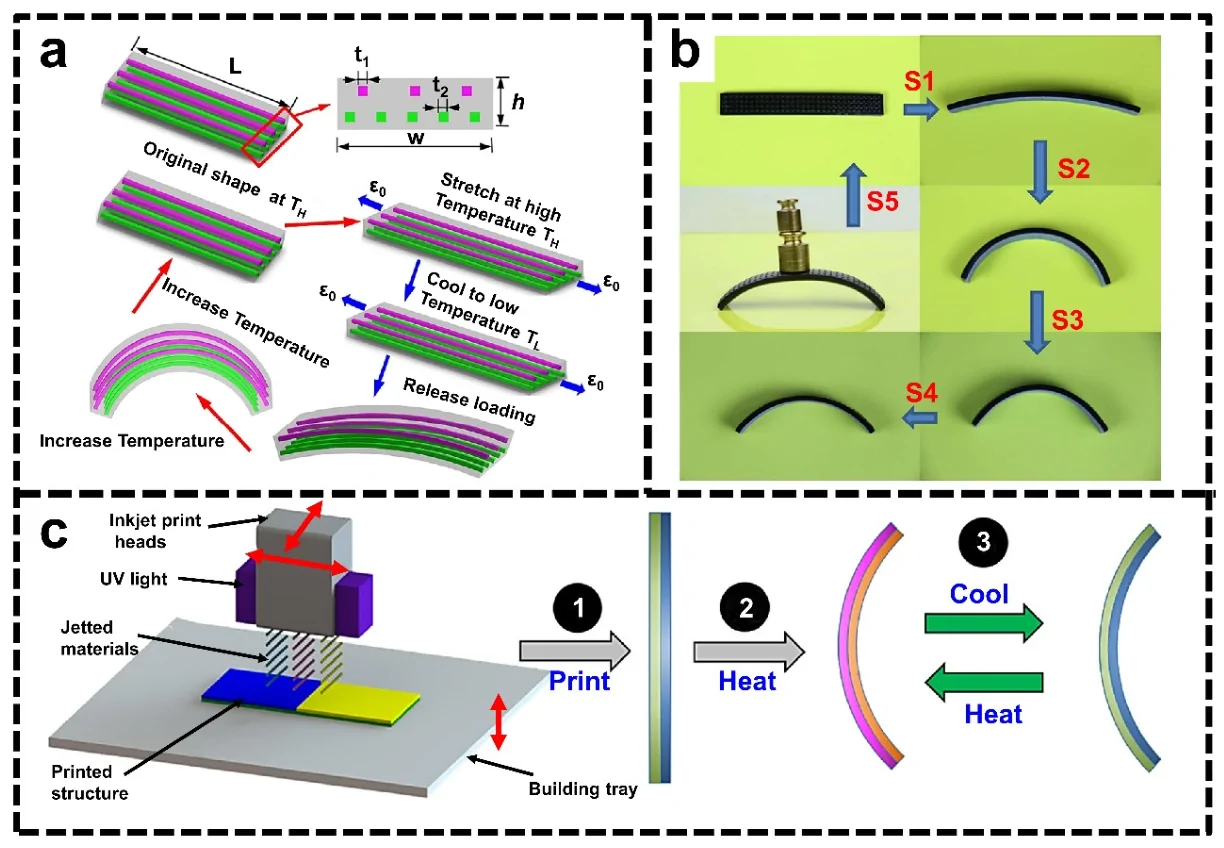
(**a**) Schematics of the printed SMP composite design and the typical programming steps for SMPs and desired response [114]. (**b**) The bending angle of two activated shape memory strips in the desired shape memory cycle [115]. (**c**) The direct 4D printing approach exploits the ability to print controlled multi-material composites. Reproduced with permission from [117]. Copyright 2017, The Authors, published by the American Association for the Advancement of Science (AAAS). Reprinted/adapted from ref. [117]. © The Authors, some rights reserved; exclusive licensee American Association for the Advancement of Science. Distributed under a Creative Commons Attribution NonCommercial License 4.0 (CC BY-NC) http://creativecommons.org/licenses/by-nc/4.0 (accessed on 27 August 2026).

Ge successfully utilized polymer jetting technology to fabricate an active material comprising an elastomer matrix reinforced with polymer fibers [113]. By strategically designing parameters such as the orientation of the polymer fibers during the printing process, the resulting printed structure demonstrated the ability to undergo autonomous deformation. Building on Ge’s research, Wu further advanced the use of polymer jetting technology to achieve 4D printing of multi-directionally deformable composite materials based on SMPs [114]. Due to the temperature-dependent viscoelastic behavior of these polymers, shape memory polymer fiber composites with varying *T*_g_ exhibited multiple shape transformations upon heating after undergoing pre-stretching, cooling and unloading processes (Figure 9a). Mao used SMPs, rubber elastomers and hydrogels as printing materials to design and successfully 4D print a bidirectionally deformable composite [115]. In this composite, the water absorption and desorption properties of the hydrogel provide the driving force for its reversible deformation, while the temperature-dependent modulus of the SMP ensures the fixation of the temporary shape. When periodic structures designed with this composite are exposed to suitable environments, they can reversibly expand and contract (Figure 9b). Teoh developed SMPs with different *T*_g_ and used polymer jetting technology to print them onto different regions of a petal [116]. Upon external heating, the petal can controllably undergo localized and overall blooming. Ding successfully 4D-printed a deformable composite using polymer jetting technology, which consisted of a two-layer structure made of SMPs and rubber elastomers [117]. Several complex 4D-printed structures were designed based on this composite, which could spontaneously deform when subjected to a thermal field without the need for additional thermal–mechanical treatment. Based on the material’s shape memory effect, the deformed structure can be reshaped again in the thermal field. Once the temporary shape is fixed, placing the structure back in the thermal field allows it to spontaneously return to its original shape (Figure 9c).

From a comparative perspective, SLA, FDM, DIW, and polymer jetting each exhibit distinct characteristics in terms of material compatibility, printing resolution, achievable structural scale, and functional integration for the 4D printing of shape memory polymers. SLA generally employs liquid photosensitive resins and is suitable for systems such as acrylates, polyurethane acrylates, epoxy acrylates, and PCL-based photocurable SMPs. With a typical printing resolution on the order of tens of micrometers, SLA is particularly advantageous for fabricating high-precision, complex microstructures and small- to medium-sized devices. SLA-printed SMPs generally exhibit high shape fixity and recovery ratios, and optimized systems can achieve values above 90%, with some approaching nearly complete recovery. However, broader applications remain constrained by the intrinsic brittleness of photosensitive resins, curing shrinkage, dependence on post-curing, and the reduced curing depth caused by light scattering from functional fillers. FDM primarily uses thermoplastic filaments as feedstock and has been widely applied to PLA, TPU, PCL, PEEK, and their composite filaments reinforced with carbon-based materials, magnetic particles, or fibers. Its typical layer thickness or printing resolution ranges from several tens to several hundreds of micrometers. Owing to its relatively low equipment cost, broad material availability, and simple processing procedure, FDM is particularly suitable for fabricating millimeter- to centimeter-scale structures and relatively large components. However, insufficient interlayer bonding, pore defects, surface roughness, and pronounced anisotropy may compromise the mechanical performance, shape recovery accuracy, and cyclic stability of the printed SMP structures. DIW uses highly viscous inks or pastes with shear-thinning and thixotropic recovery behavior, and is suitable for epoxy, PLA solution, SMPU, hydrogels, LCEs, and highly filled conductive, magnetically responsive, and photothermal composite systems. Its resolution is mainly governed by nozzle diameter and ink rheology and can range from hundreds of micrometers to approximately 20 μm. DIW offers clear advantages in material selection, incorporation of functional fillers, and control of fiber or liquid crystal orientation. Shape memory performance commonly exceeds 90%, and some nanocomposite systems have reported shape recovery or fixity values of approximately 94–99%. However, printing speed and forming stability are strongly constrained by ink viscosity, curing rate, solvent evaporation, nozzle clogging, and structural collapse. By contrast, polymer jetting forms structures by jetting low-viscosity photopolymer droplets followed by in situ UV curing. Its resolution is typically in the tens-of-micrometers range, and it is suitable for multi-material structures, gradient-*T*_g_ designs, SMP/elastomer/hydrogel composites, and locally programmable deformation. It offers higher forming efficiency and multi-material integration than single-nozzle path-based printing, especially for complex small devices and reversible or sequentially deforming structures. However, its material window is relatively narrow, usually relying on proprietary low-viscosity photopolymer resins. Equipment cost, filler loading capacity, interfacial reliability, long-term mechanical stability, and large-scale manufacturing remain major limitations. Therefore, SLA is more suitable for high-precision photocurable SMP devices, FDM for low-cost and large thermoplastic SMP structures, DIW for highly filled, multifunctional, and orientation-programmable composite systems, and polymer jetting for multi-material, highly integrated, and spatially graded deformation designs. It should be noted that resolution, printing efficiency, and shape memory performance reported in different studies are strongly affected by material composition, printing equipment, specimen geometry, post-treatment conditions, and testing methods.

## 4. Applications of 3D/4D-Printed Shape Memory Polymers

### 4.1. Biomedical Field

**Figure 10:**
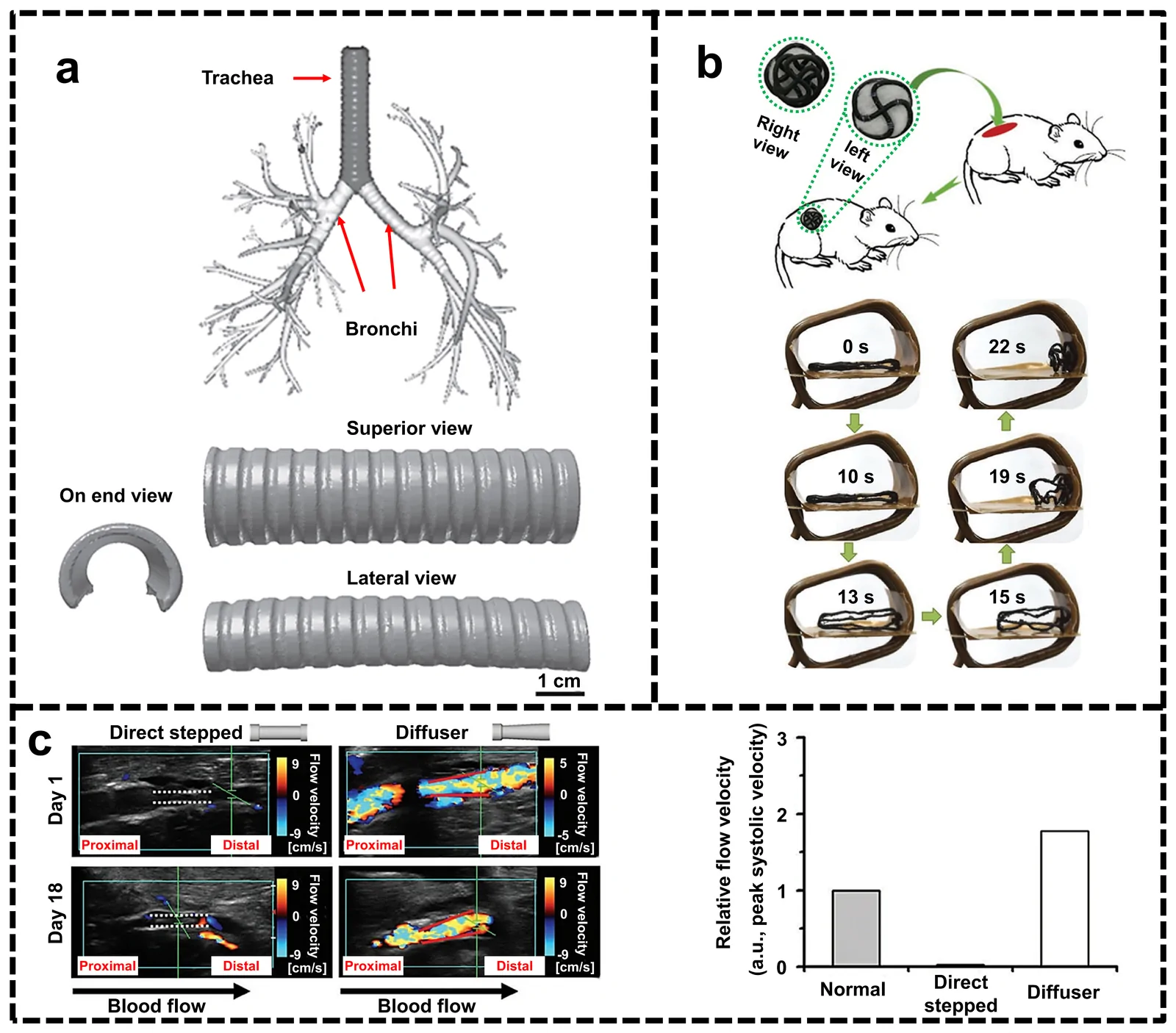
(**a**) Fabrication procedure of the shape memory airway stent. Reproduced with permission from [123]. Copyright 2016, John Wiley and Sons. (**b**) Schematic illustration of the implantation process (**up**) and programmed shape recovery process of a 10 wt% mPLA 4-arm occluder under a magnetic field (**down**). Reproduced with permission from [124]. Copyright 2019, John Wiley and Sons. (**c**) Doppler ultrasound imaging assessment of graft patency with live monitoring of pulsation synchronization in the video (**left**) and blood flow velocity inside SMP vascular grafts at day 18 after implantation with normalization based on the velocity inside the normal femoral artery without grafting (**right**). Reproduced with permission from [126]. Copyright 2019, John Wiley and Sons.

4D-printed SMPs that can adapt and respond to the human body’s internal environment, while allowing for personalized structural customization, have significant potential applications in minimally invasive medical devices and tissue engineering scaffolds [118,119,120,121,122]. Zarek used digital light processing technology to print a tracheal stent made from a light-crosslinked polycaprolactone methyl methacrylate polymer [123]. When heated to 40–50 °C, the stent softens and can be curled under external force to reduce its volume, facilitating smaller incisions during implantation. After implantation, the stent undergoes self-expansion when exposed to thermal stimulation, thereby enlarging the trachea (Figure 10a). Lin prepared an occluder by incorporating Fe_3_O_4_ magnetic particles into a shape memory polylactic acid matrix [124]. The occlude exhibits excellent cytocompatibility and histocompatibility, facilitating cell adhesion and the ingrowth of granulation tissue, thereby promoting rapid endothelialization (Figure 10b). Zhang printed SMP scaffolds adapted for biomedical implants, studying the shape memory and thermomechanical properties of these SMP scaffolds [125]. The scaffolds exhibited excellent cyclic shape memory performance and the ability to rapidly return to their original shape. Shin successfully transplanted shape memory tubes into pig femoral arteries with diameters smaller than 3 mm [126]. Utilizing their shape memory effect, these tubes effectively prevented thrombus-induced physical blockage of the blood vessels (Figure 10c). Zema developed a gastric retention drug delivery system (GRDDS) based on shape memory PVA material using FDM technology [127]. By placing the printed structure inside a gelatin capsule, the solvent-driven shape memory effect allows the GRDDS to interact with gastric fluid at body temperature and release the drug in a time-dependent manner.

**Figure 11:**
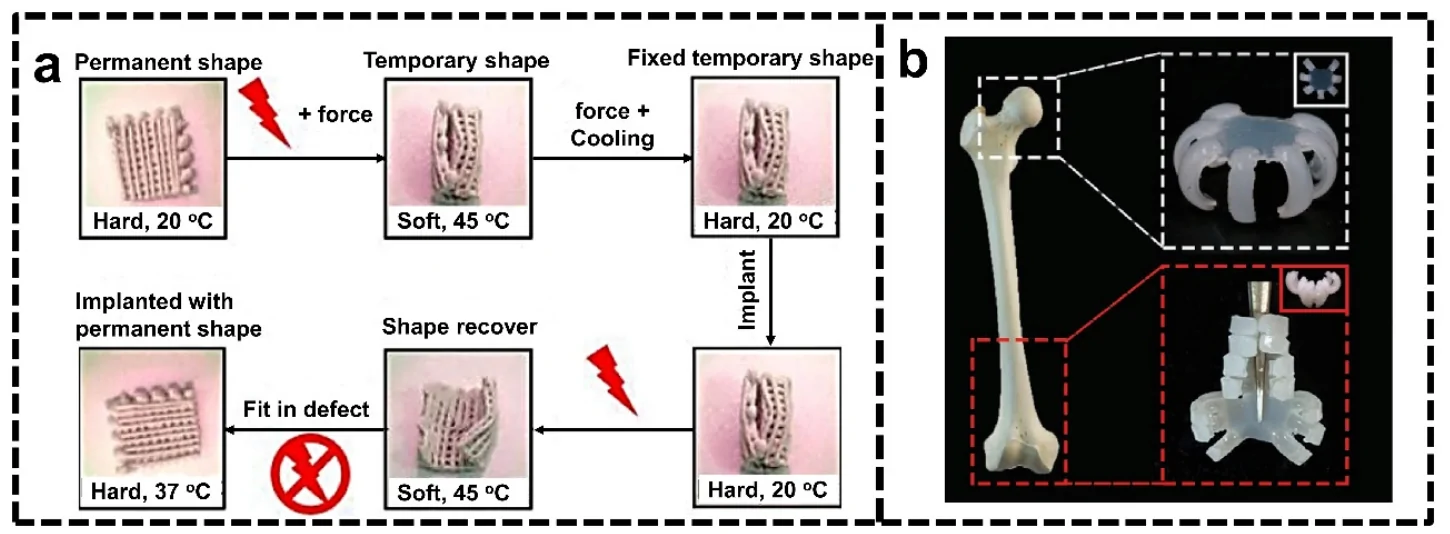
(**a**) Shape memory cycle of BPTS scaffolds, from original shape to deformed shape and further to recovered shape. Used with the permission of the Institute of Physics, from [Advanced reconfigurable scaffolds fabricated by 4D printing for treating critical-size bone defects of irregular shapes, Institute of Physics, 12(4) (2020)] [129]. (**b**) Custom-fitting of 4D-printed bilayer membranes. Reproduced with permission [131]. Copyright 2021, John Wiley and Sons.

Cheng prepared an elbow protector based on an NW-PLA-PCL network using ultraviolet-assisted FDM printing [128]. When the temperature was reduced to 55 °C, the material demonstrated a fixation rate and shape recovery rate exceeding 91%, highlighting its excellent printability, mechanical properties and shape memory performance. Wang developed a light- and heat-driven shape memory bone tissue engineering scaffold using low-temperature 4D printing for the treatment of irregular bone defects [129]. The scaffold’s ability to respond to light and heat allowed for easy implantation into irregularly shaped bone defects via narrow scaffold channels, while maintaining high compressive strength throughout the bone regeneration process (Figure 11a). Zhang et al. developed a physiologically adaptive 4D-printed hydrogel smart cardiac patch for the treatment of myocardial infarction [130]. Compared to the untreated group, the treatment group exhibited higher cell density and smaller necrotic areas, demonstrating the therapeutic effectiveness of the 4D-printed patch. You fabricated a bilayer, multi-responsive composite film based on a shape memory layer of polycaprolactone-diacrylate (PCLDA) and a hydrogel layer [131]. The surface microstructure of the shape memory layer could be precisely switched under external stimuli, promoting osteoblast proliferation and differentiation at different stages. The hydrogel layer enabled the composite film to adjust its three-dimensional shape, allowing it to conform to specific bone structures in clinical applications. With this bilayer multi-responsive composite film, new bone formation increased by 30% (Figure 11b). Hwangbo fabricated a biomimetic collagen/hydroxyapatite composite bone scaffold with multiple internal microchannels [132]. This scaffold mimics the layered, porous structure of bone and provides an ideal environment for osteogenic differentiation of osteoblasts. The internal microchannel network not only increases the scaffold’s specific surface area but also enhances its physiological properties, such as capillary stress. These features promote osteogenic differentiation and angiogenesis of cells on the scaffold, facilitating bone tissue regeneration and repair at the defect site. Choudhury et al. used a single PLMC shape memory thermoplastic polymer and controlled printing infill angle and structural anisotropy to roll two-dimensional sheets into hollow tubular structures with diameters of approximately 6–10 mm and lengths up to 40 mm within 1 min at approximately 80 °C, followed by shape memory recovery near 37 °C for the construction of endothelialized vascular grafts [133]. In addition, Choudhury et al. used PLMC as the SMP matrix, incorporated photothermal silver nanoparticles, and further loaded Pheophorbide-a, enabling the material to combine NIR-triggered shape recovery, fluorescence imaging, and photoacoustic imaging. The 3D-printed scaffolds and stent-like structures achieved more than 99% shape recovery under near-physiological temperature conditions and showed potential for multimodal non-invasive imaging [134]. In addition to biomedical devices based on classical shape memory polymers (SMPs), hydrogel-based 4D-printed structures have attracted increasing attention because of their high water content, tissue-like softness, and good cytocompatibility. Joshi et al. showed that by designing hydrogel swelling behavior and printed infill patterns, 3D-printed hydrogel sheets could rapidly self-roll in vivo into tubular nerve conduits for sutureless repair of rat sciatic nerve defects, with nerve regeneration verified by histological and functional evaluations. Compared with hydrogel-based devices, classical shape memory polymers generally offer higher mechanical strength, better dry-state shape fixity, and improved dimensional stability, which are particularly important for deployable implants requiring structural support and long-term shape retention [135].

### 4.2. Flexible Electronics Field

**Figure 12:**
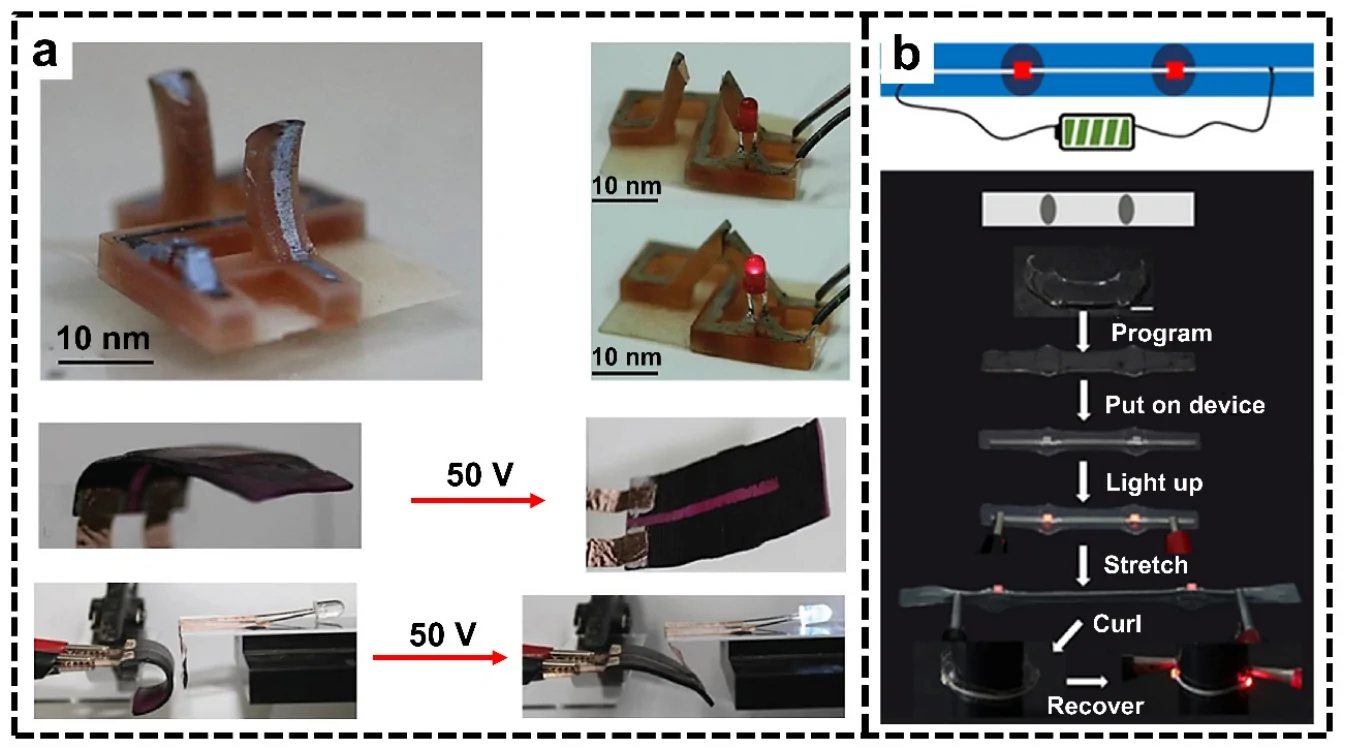
(**a**) Fabrication of shape memory-based electrical devices. Reproduced with permission from [93]. Copyright 2015, John Wiley and Sons. (**b**) Demonstration of the flexible wearable device substrate. Reproduced with permission from [140]. Copyright 2019, American Chemical Society.

Traditional manufacturing methods typically allow electronic devices to be processed into two-dimensional or simple three-dimensional shapes [136,137]. However, 4D printing of SMPs and their composites enables the creation of advanced smart electronic devices [138,139]. Zarek developed a deformable temperature sensor by inkjet printing conductive silver paste onto a 3D-printed polycaprolactone methacrylate (PCLMA) structure created using digital light processing technology [93]. When the sensor is exposed to a thermal environment above its melting temperature, it returns to its original shape, reconnecting the conductive circuit and forming an electrical path. This process triggers an LED to light up, signaling the warning temperature. Furthermore, by depositing carbon nanotubes onto a flat panel 4D-printed with the same material, an electrically driven switch was fabricated. When a 50 V voltage is applied, the “U”-shaped carbon nanotube layer deposited on the flat panel serves as an electric heater, generating heat that stimulates the pre-bent panel to spontaneously return to its initial flat state. This action closed the circuit and lights the LED connected to it, thus functioning as a switch (Figure 12a). Zhang used methyl methacrylate and isobornyl acrylate as copolymer monomers and 1,6-hexanediol diacrylate as a crosslinker [140]. By precisely controlling the 3D structure fabrication through digital light-curing technology, a 3D structure made of multiple SMPs was printed from the same precursor ink. This structure was then applied to retractable flexible electronic products (Figure 12b).

**Figure 13:**
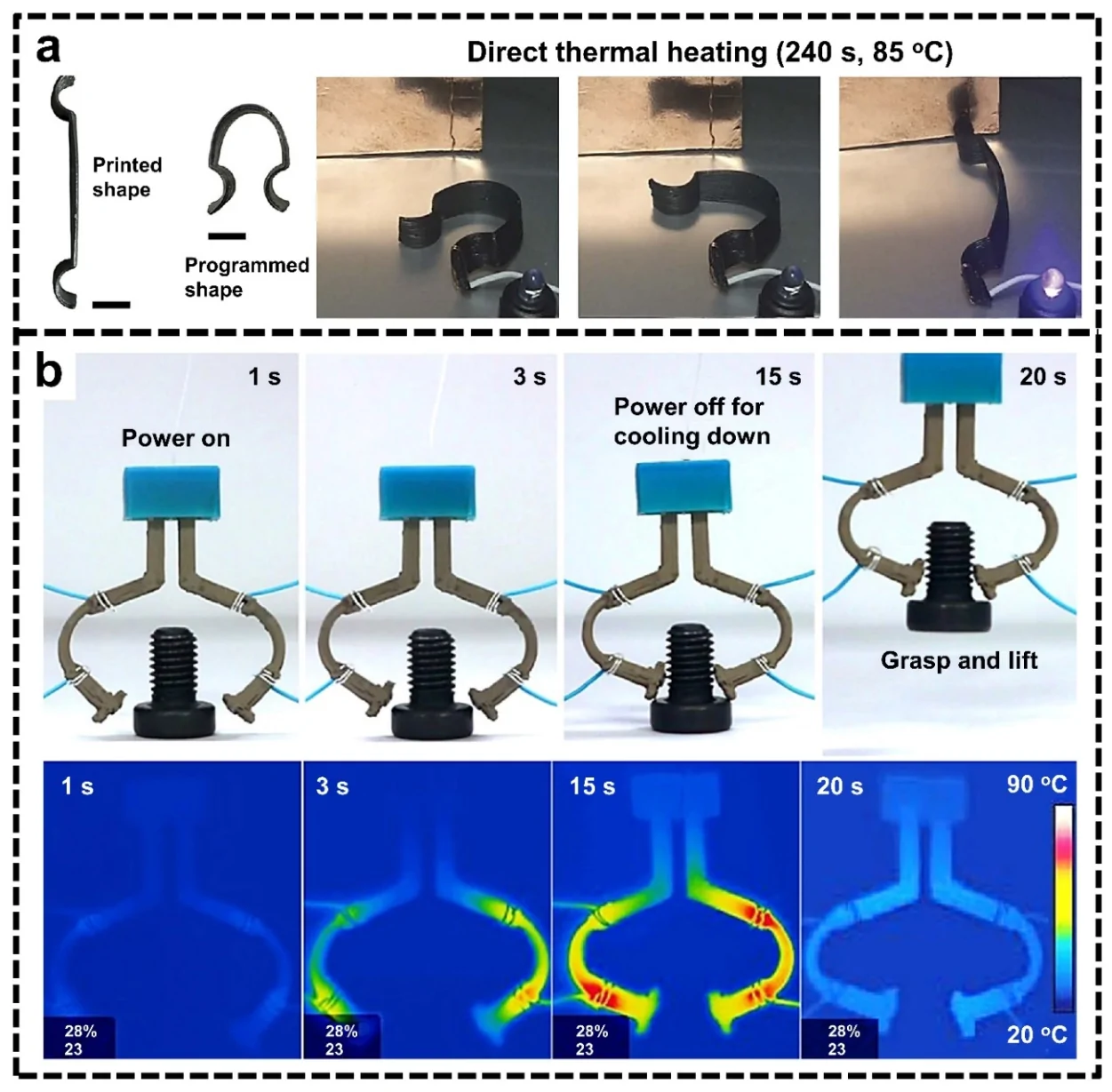
(**a**) An electrically conductive (5.6 vol% CNF) part is shown in its as-printed or primary shape and its programmed shape. The part from (left) is connected to a power source and thermally actuated to change its shape and complete a circuit in (right) where an LED becomes powered and lights up after 240 s at 80 °C [142]. (**b**) The electrical-actuated shape recovery process of an electroactive smart gripper. Reproduced with permission from [143]. Copyright 2019, American Chemical Society.

Bodkhe prepared a 4D printing ink using shape memory PLA, polyamide and barium titanate nanoparticles as raw materials, with dichloromethane as the solvent. Using direct-write 3D printing, a deformable piezoelectric sensor was fabricated [141]. The sensor exhibited a linear voltage response to external forces ranging from 0.1 N to 1 N and could withstand over 5000 operating cycles. Rodriguez fabricated a thermoset SMP deformable conductive component using DIW printing [142]. The printing ink was mixed with carbon nanofibers (CNFs) to enable the electrically driven shape memory effect of the SMP. The thermal-driven shape memory effect of the conductive component activated the circuit, successfully lighting up an LED after 240 s at 80 °C (Figure 13a). Wei fabricated high-performance electrical devices based on high-conductivity SMPCs through 4D printing, including microstructured fiber sensors, flexible supports for electromagnetic shielding and low-voltage-triggered smart grippers [143]. The SMPC, which incorporates silver-coated carbon nanofibers, exhibits a low penetration threshold and high electrical conductivity, demonstrating fast and low-voltage-triggered electrically driven shape memory behavior, making it an ideal choice for electrically driven smart devices (Figure 13b).

### 4.3. Soft Robotics

SMPs and their composites exhibit not only shape transformation upon heating but also a corresponding variation in their mechanical modulus. This characteristic, which is closely linked to changes in material properties with temperature, endows 4D-printed structures derived from SMPs and their composites with considerable potential for applications in advanced smart systems, particularly in the development of adaptive grippers.

**Figure 14:**
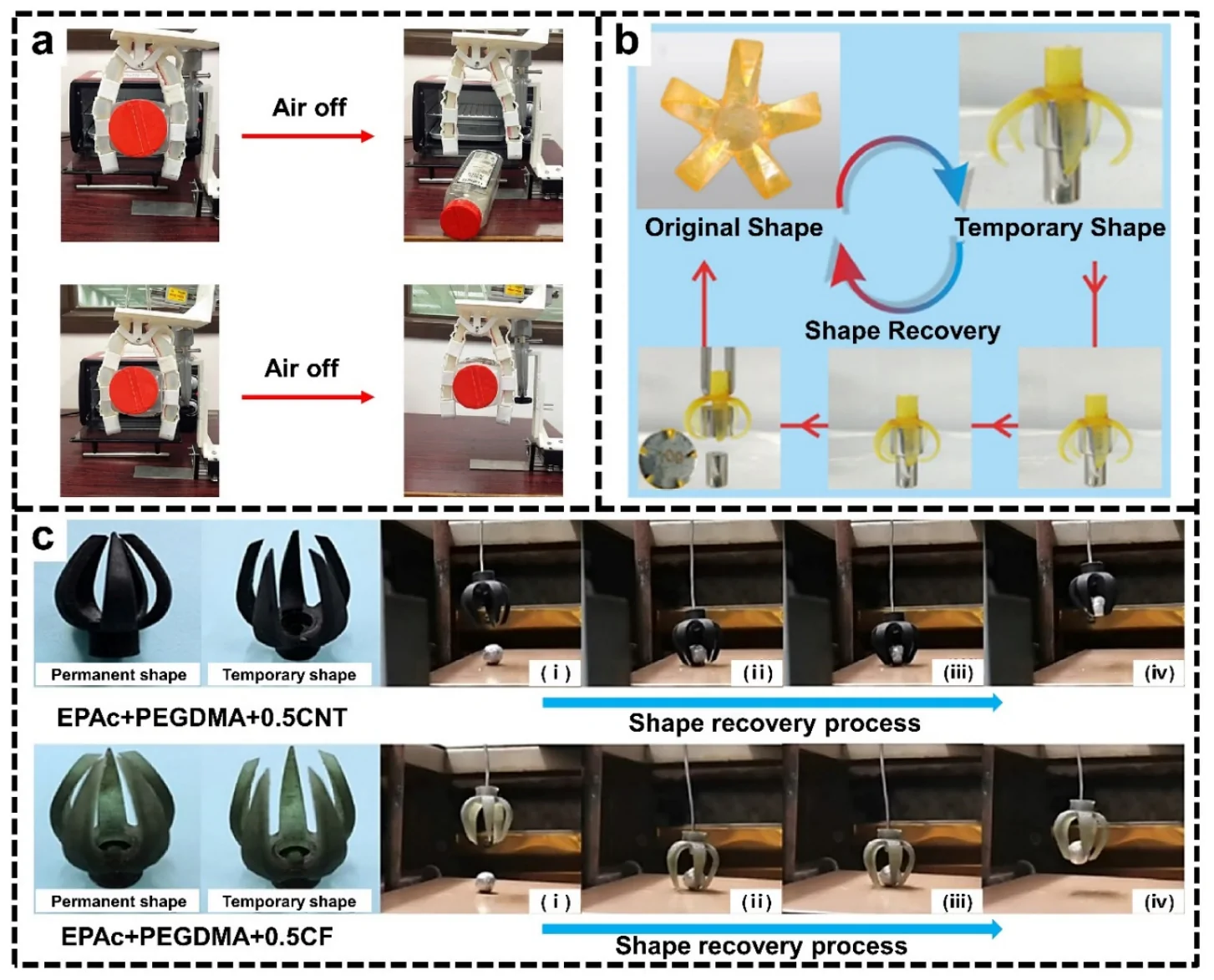
(**a**) Grasping experiments of a robotic hand or gripper. Used with the permission of the American Society of Mechanical Engineers, ASM International, from [Novel Design and Three-Dimensional Printing of Variable Stiffness Robotic Grippers, American Society of Mechanical Engineers., ASM International, 8(6) (2016)] [144]. (**b**) Process of grabbing weight of 10 g (IEMSi3). Reproduced with permission from [145]. Copyright 2019, American Chemical Society. (**c**) Shape recovery process of printed capture devices based on EPAc + PEGDMA + 0.5 CNT resin (**up**) and EPAc + PEGDMA + 0.5 CF resin (**down**). Reproduced with permission from [146]. Copyright 2021, American Chemical Society.

**Figure 15:**
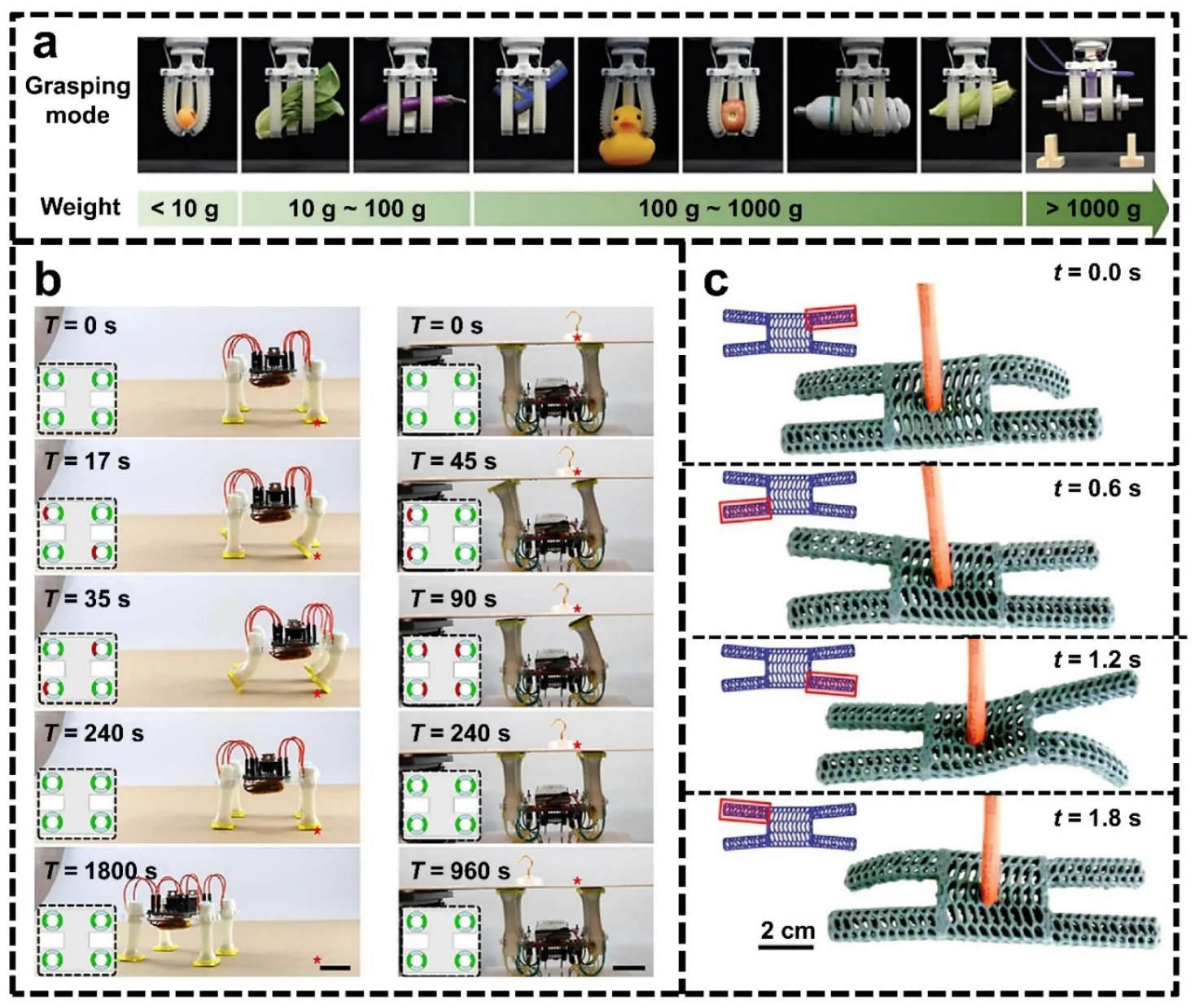
(**a**) Demonstration of high load capacity and good shape adaptivity by a versatile gripper equipped with three FRST actuators. Grasping of objects with arbitrary shapes and various weights ranging from less than 10 g to 1.5 kg. Reproduced with permission from [147]. Copyright 2019, John Wiley and Sons. (**b**) Frames from a video of the electrically activated, untethered soft robot walking (**left**) and frames from the video of the robot transporting an object on a plate of cardboard (**right**). Reproduced with permission from [148]. Copyright 2019, The Authors, published by the American Association for the Advancement of Science (AAAS). Reprinted/adapted from ref. [148] © The Authors, some rights reserved; exclusive licensee American Association for the Advancement of Science. Distributed under a Creative Commons Attribution NonCommercial License 4.0 (CC BY-NC) http://creativecommons.org/licenses/by-nc/4.0 (accessed on 27 August 2026). (**c**) A basic crawling gait of ASM quadruped. Reproduced with permission from [149]. Copyright 2019, John Wiley and Sons.

Yang fabricated an SMP and used the fused deposition technique to print a biomimetic smart gripper [144]. When the temperature exceeds the *T*_g_ of the SMP, the joints undergo rapid softening. This allows the fingers, powered by a pneumatic actuator, to bend. Upon cooling, the modulus of the joints increases and the bent state is locked in, enabling the gripper to actively grasp and manipulate objects (Figure 14a). Miao prepared a novel methacrylate material containing dynamic imine bonds (IEMSis) using a new aldehyde-functionalized (meth)acrylate monomer and a hyperbranched crosslinker (HPASi) [145]. Compared with the material without HPASi, the flexible chain structure of HPASi increased the toughness of IEMSis by 33 to 97 times. Moreover, the incorporation of HPASi endowed IEMSis with excellent shape memory properties, with shape fixity and shape recovery ratios reaching 97.5–97.6% and 91.4–93.7%, respectively (Figure 14b). Leng first synthesized an epoxy acrylate through an esterification reaction between epoxy resin and acrylic acid [146]. Subsequently, a fast photocurable composite ink was developed by incorporating a crosslinking agent, polyethylene glycol dimethacrylate and reinforcing carbon fillers. Using this ink, a claw-shaped capturing device was printed, which demonstrated the ability to grasp a tin foil ball due to the excellent shape memory properties of the material (Figure 14c). Zhang designed a fast-response gripper by embedding an SMP layer into a 3D-printed robotic claw [147]. The gripper, with adjustable stiffness, can lift objects ranging from 10 g to 1.5 kg within 32 s (Figure 15a). He created a remote-controlled multimodal soft robot by integrating LCE materials with a microcontroller [148] (Figure 15b). Goswami used flexible photopolymers and SLA technology to print a low-density soft robot [149]. The deformation and movement of the soft robot, including contraction, twisting, bending and cyclic motion, are dependent on its topology—such as the type of lattice or tessellation, beam thickness and the size of the tessellated units (Figure 15c).

## 5. Conclusions and Future Perspectives

This review has summarized the material basis, printing strategies, actuation routes, and representative applications of SMP-based 4D printing. Compared with static 3D-printed polymers, 4D-printed SMP structures can store a programmed temporary geometry and recover a designed shape when exposed to appropriate stimuli. The field has advanced rapidly in thermal, electrical, magnetic, optical, and solvent-driven systems, and these approaches have enabled demonstrations in biomedical devices, soft robotics, flexible electronics, and deployable structures.

Although 4D-printed SMPs and their composites have shown broad promise in programmable structures, soft actuators, flexible electronics, and biomedical devices, their transition from laboratory proof-of-concept demonstrations to industrial applications still faces multiple barriers. First, there is an intrinsic trade-off between material performance and printability. Improving shape recovery ratio, response rate, mechanical strength, or multi-stimulus responsiveness usually requires increasing crosslinking density, regulating crystalline structure, or introducing conductive, magnetic, or photothermal functional fillers. However, these modifications may increase melt or ink viscosity, cause nozzle clogging, reduce photocuring depth, promote filler aggregation, introduce interfacial defects, and embrittle the material. Second, manufacturing consistency and scalability still need improvement. Pores, insufficient interlayer bonding, and path-dependent anisotropy in FDM-printed parts, curing shrinkage, residual stress, and filler-induced light scattering in SLA/DLP systems, rheological instability, solvent evaporation, and structural collapse in DIW inks, and the long-term reliability of multi-material interfaces in polymer jetting can all affect dimensional accuracy and performance repeatability in batch manufacturing. Third, cyclic stability and long-term service reliability remain insufficiently evaluated. Many studies mainly verify a single or only a few shape memory cycles, whereas practical applications require materials to maintain stable shape fixity ratio, shape recovery ratio, recovery time, output force, and mechanical integrity during repeated programming, storage, triggering, and recovery. Finally, differences in specimen geometry, printing parameters, stimulus conditions, testing environment, and evaluation metrics among current studies limit direct comparison across material systems and printing processes. In addition, although 4D-printed SMPs in biomedical applications show promise in scaffolds, vascular grafts, drug delivery systems, and minimally invasive implantable devices, in vitro test results cannot be directly extrapolated to in vivo performance. Temperature fluctuations, water diffusion, enzymatic degradation, protein adsorption, immune responses, tissue constraints, degradation-product toxicity, and sterilization processes in vivo may all alter the transition temperature, mechanical properties, and shape recovery behavior of the material. Multi-material 4D printing can enable gradient modulus, gradient *T*_g_, local response, and sequential deformation, and is an important route for constructing complex programmable SMP devices. However, mismatches in viscosity, curing rate, thermal expansion, shrinkage behavior, and mechanical modulus among different materials can lead to insufficient interfacial bonding, warping, cracking, delamination, and long-term cyclic failure.

Future work should therefore move beyond isolated improvements in either materials or printing processes and instead adopt an integrated strategy involving material design, structural programming, process optimization, and predictive modeling. Copolymerization, polymer blending, dynamic crosslinked networks, and epoxy–acrylate hybrid resin systems provide useful routes to regulate transition temperature, cross-link density, toughness, curing shrinkage, and shape memory performance. For composite systems, surface modification of fillers, optimization of particle size and loading content, reduction in the percolation threshold, and improvement of matrix–filler interfacial compatibility are essential for enhancing actuation efficiency while minimizing aggregation, viscosity increase, and curing inhibition. At the structural and processing levels, multi-material printing, gradient architectures, toolpath design, controlled fiber or filler orientation, post-curing, and thermal treatment can be used to improve interlayer adhesion, deformation controllability, and shape recovery stability. In parallel, thermomechanical–viscoelastic–phase transition coupled models are needed to link printing parameters, material composition, structural geometry, and shape memory behavior. Such models will be critical for moving 4D-printed SMPs from empirical demonstrations toward predictable design and reliable engineering applications.

## Figures and Tables

**Table 1:** Comparison of this review with previous representative reviews.

Representative Previous Reviews	Main Focus	Difference from This Review	Added Value of This Review
Additive manufacturing-A review of 4D printing and future applications [34]	General 4D printing materials, structures, and applications	Does not specifically focus on SMPs and rarely compares SMP actuation mechanisms and printing compatibility in depth	This review focuses on 4D-printed SMPs and emphasizes the relationships among shape memory mechanisms, stimulus responsiveness, and printing processes
A Review of 3D Printing Technologies for Soft Polymer Materials [35]	3D printing technologies for soft polymers	Focuses on 3D printing of soft materials rather than 4D-printed SMPs	This review further discusses the shape fixing/recovery mechanisms of SMPs and their 4D printing applications
Fused Filament Fabrication-4D-Printed Shape Memory Polymers: A Review [36]	FFF/FDM-printed SMPs	Mainly focuses on a single extrusion-based printing technique	This review compares SLA/DLP, FDM/FFF, DIW, and polymer jetting
4D printing of shape memory polymer composites: A review on fabrication techniques, applications, and future perspectives [37]	SMPC fabrication technologies and applications	Emphasizes composite materials and application overviews, with insufficient cross-comparison of stimulus modes and biomedical compatibility	This review adds a comparison of different stimuli in terms of response time, efficiency, spatial control, and biomedical applicability
Liquid-Based 4D Printing of Shape Memory Nanocomposites: A Review [38]	Liquid-based systems and nanocomposite SMPs	Mainly focuses on liquid-based printing and nanocomposite systems	This review covers thermoplastic, photocurable, ink-based, and multi-material-jetted SMP systems
A Review of Shape Memory Polymers and Composites: Mechanisms, Materials, and Applications [13]	SMP/SMPC mechanisms, materials, and applications	Focuses on SMP materials themselves rather than using 4D printing processes as the main organizing framework	This review links SMP mechanisms with 4D printing processes, structural programming, and application scenarios

**Table 2:** Comparison of different stimuli in terms of response time, actuation efficiency, spatial controllability, and biomedical compatibility.

Stimulus	Response Time	Actuation/Recovery Efficiency	Spatial Controllability	Biomedical Compatibility	Main Advantages	Main Limitations
Thermal	Usually seconds to minutes; slower in large or thick-walled structures because of limited heat conduction	Usually high; optimized systems can achieve more than 90% shape recovery	Low to moderate; mainly based on bulk heating, and regional selectivity requires local heating, electrothermal, or photothermal designs	Depends on transition temperature; systems triggered near body temperature or at low temperature are more suitable, whereas excessive temperature may cause thermal tissue damage	Mature mechanism, broad material applicability, and high recovery efficiency	Slow heat diffusion, limited local control, and possible risk of thermal damage
Electrical	Usually seconds to minutes	High; once conductive networks are formed, recovery can be rapidly triggered by Joule heating, and some systems can achieve nearly complete recovery	High; local or selective actuation can be achieved through electrode layout, conductive pathways, and printing paths	Moderate to low; more suitable for in vitro or wearable scenarios, whereas in vivo applications are limited by voltage, electrodes, local overheating, and conductive-filler safety	Fast response, easy integration with flexible electronics, and strong local actuation capability	Requires conductive fillers or conductive networks; high voltage and Joule heating may introduce safety risks
Magnetic	Usually seconds to minutes	High; depends on magnetic filler content, dispersion, and magnetothermal conversion efficiency	High; remote or local regulation can be achieved through magnetic particle distribution, content gradients, magnetic field direction, and field strength	Materials such as Fe_3_O_4_ have a basis for biomedical use, but particle leakage, dose, field strength/frequency, and local overheating must be controlled	Non-contact and remote triggering with relatively strong tissue penetration; suitable for implants and minimally invasive devices	Fillers may affect mechanical properties and printability; magnetic field equipment requirements are high, and long-term safety still requires evaluation
Optical	Usually seconds to minutes	Moderate to high; depends on photothermal fillers, light intensity, irradiation time, and light penetration depth	Very high; region-selective deformation can be achieved through local irradiation, masks, wavelength selection, and filler patterning	Depends on wavelength; near-infrared light is more suitable for biomedical applications, whereas ultraviolet light is limited by low penetration and potential damage	Non-contact activation with high spatial resolution; suitable for precise local actuation	Limited light penetration depth restricts deep-tissue applications; the biosafety of photothermal fillers requires evaluation
Water/solution	Usually minutes to hours; thin-walled hydrogels may shorten the response to the hundred-second range	Moderate to high; depends on swelling, plasticization, hydrogen-bond reconstruction, or diffusion processes	Moderate; some control can be achieved through material distribution, structural thickness, and local swelling design, but localization is less precise than optical or electrical stimulation	High; suitable for hydrogels, PVA, biodegradable polymers, drug delivery, and body-fluid environments	Mild triggering under near-physiological conditions; suitable for biomedical scenarios	Slow response, limited output force, and dimensional and cyclic stability that are sensitive to water content

## Data Availability

No new data were created or analyzed in this study. Data sharing is not applicable to this article.
